# A Super‐Enhancer‐Driven Transcriptional Regulatory Circuit Underlying Abiraterone Resistance in Castration‐Resistant Prostate Cancer

**DOI:** 10.1002/advs.202501284

**Published:** 2025-06-05

**Authors:** Liling Jiang, Jiamin Wang, Guanjie Peng, Haichuan Zhang, Jinxin Fang, Yingyin Gao, Enzhe Lou, Yangzhou Liu, Wa Ding, Bingyuan Liu, Qiong Mao, Lizhen Jiang, Aochu Liu, Xinyue Li, Shiwen Hu, Qiaomin Ma, Yueyuan Zheng, Zhigang Zhao, Xianping Shi

**Affiliations:** ^1^ Guangzhou Municipal and Guangdong Provincial Key Laboratory of Protein Modification and Degradation Sino‐French Hoffmann Institute School of Basic Medical Sciences Guangzhou Medical University Guangzhou Guangdong 511436 P. R. China; ^2^ The Affiliated Traditional Chinese Medicine Hospital Guangzhou Medical University Guangzhou Guangdong 510130 P. R. China; ^3^ Department of Urology Guangdong Provincial Key Laboratory of Major Obstetric Diseases Guangdong Provincial Clinical Research Center for Obstetrics and Gynecology The Third Affiliated Hospital Guangzhou Medical University Guangzhou Guangdong 510150 P. R. China; ^4^ Clinical Big Data Research Center Scientific Research Center The Seventh Afffliated Hospital of Sun Yat‐sen University Shenzhen Guangdong 518107 P. R. China; ^5^ Department of Urology The First Affiliated Hospital Guangzhou Medical University Guangzhou Guangdong 510120 P. R. China

**Keywords:** abiraterone resistance, castration‐resistant prostate cancer, transcriptional regulation

## Abstract

Castration‐resistant prostate cancer (CRPC) remains the leading cause of mortality among prostate cancer patients. While second‐generation androgen receptor (AR) pathway‐targeted therapies, such as Abiraterone, have significantly improved survival outcomes, resistance to these treatments ultimately emerges, posing a critical challenge. Understanding the mechanisms underlying Abiraterone resistance is essential for developing strategies to enhance patient outcomes. In this study, a super‐enhancer (SE)‐driven transcriptional regulatory circuit is identified involving BCL6, NFIB, and SMAD3 that facilitates Abiraterone resistance in CRPC. Through comprehensive analyses of SE expression profiles in Abiraterone‐resistant CRPC cells and their parental counterparts, it is revealed that this circuit plays a pivotal role in resistance progression. Mechanistically, BCL6, NFIB, and SMAD3 synergistically remodel the transcriptional landscape of resistant CRPC cells, driving resistance by regulating cholesterol biosynthesis and cell cycle pathways. The findings provide critical insights into the transcriptional dysregulation underlying Abiraterone resistance and highlight potential therapeutic strategies to counteract treatment resistance in CRPC, ultimately aiming to improve patient survival and quality of life.

## Graphical abstract

This study identifies a super‐enhancer‐driven transcriptional regulatory circuit comprising BCL6, SMAD3, and NFIB that cooperate to drive cholesterol synthesis via SREBF2/HMGCR/FDFT1 activation and regulate CDK2/CCND3 for cell cycle control. Targeting this circuit with BI‐3802 or downstream inhibitors (Fatostatin/Lovastatin) overcomes abiraterone resistance in CRPC.

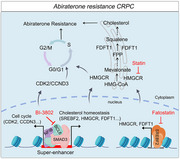

## 1 Introduction

Prostate cancer (PCa) is the most common tumor and the second leading cause of death in men in the United States and other Western countries.^[^ ^1^ ^]^ Androgen deprivation therapy (ADT) is the main method for the treatment of advanced prostate cancer. However, after an average of 2 years of ADT treatment, the tumor often progresses from castration‐sensitive prostate cancer (CSPC) to castration‐resistant prostate cancer (CRPC) with a poor prognosis.^[^ ^2^ ^]^

Abiraterone has been approved by the FDA as a first‐line treatment for metastatic castration‐resistant prostate cancer (mCRPC) both before chemotherapy and in cases where chemotherapy has failed. It functions by inhibiting CYP17A1, an enzyme critical for androgen precursor synthesis, targeting 17α‐hydroxylase and 17,20‐lyase, thereby impeding androgen production.^[^ ^3^ ^]^ However, the development of acquired resistance to Abiraterone significantly compromises treatment efficacy and patient survival. Studies have demonstrated an association between androgen receptor (AR) expression/activity and anti‐androgen resistance in CRPC.^[^ ^4^, ^5^, ^6^, ^7^ ^]^ Aberrant expression and splice variants of the *AR* gene are implicated in resistance mechanisms. The AR‐V7 androgen receptor splice variant is currently the most well‐defined resistance variant, lacking the androgen‐binding domain but retaining an activation domain that promotes tumor growth.^[^ ^8^, ^9^ ^]^ In light of this, scholars are striving to identify new therapeutic targets and develop more effective drugs to eradicate CRPC, such as AR‐V7 degraders and the AKT inhibitor Ipatasertib.^[^ ^10^, ^11^ ^]^ Nevertheless, the efficacy of these new treatments remains to be definitively established, and resolving Abiraterone resistance in CRPC patients is an urgent priority.

Super‐enhancers (SEs) are large clusters of transcriptional enhancers characterized by exceptionally high levels of histone modifications associated with active transcription, such as H3K27ac and H3K4me1, as well as dense binding of transcription factors and mediator complex subunits.^[^ ^12^, ^13^ ^]^ SEs recruit a number of transcriptional activators, such as histone modification enzymes, transcription factors and cofactors, exhibiting stronger transcriptional activation capabilities than typical enhancers.^[^ ^14^ ^]^ In contrast to typical enhancers, SEs orchestrate the expression of lineage‐defining genes by forming spatially organized 3D chromatin loops that facilitate physical interactions between distal regulatory elements and target promoters.^[^ ^15^ ^]^ Notably, SEs frequently govern cell identity programs and are enriched for disease‐associated variants, highlighting their pathological relevance in cancer and genetic disorders.^[^ ^16^ ^]^ Researches suggest that transcriptional regulatory loops driven by super‐enhancers promote tumorigenesis. For example, in T‐cell acute lymphoblastic leukemia, TAL1, GATA3 and RUNX1 form a core transcriptional regulatory loop that activates the *MYB* gene, enhancing the regulation of oncogenic programs.^[^ ^17^ ^]^ In multiple myeloma, SEs drive the formation of a transcriptional regulatory network involving transcription factors from the IRF, ETS, MEF2, E‐box and AP‐1 families, collaboratively promoting the malignant phenotype of the tumor.^[^ ^18^ ^]^ Tumor types that have been studied in association with core transcriptional regulatory loops also include esophageal squamous cell carcinoma, esophageal adenocarcinoma and neuroblastoma.^[^ ^19^, ^20^ ^]^ Furthermore, our research group previously elucidated the mechanism by which a SE‐driven core transcriptional regulatory loop composed of TCF4, KLF15 and NKX2.2 promotes the initiation and progression of Ewing sarcoma.^[^ ^21^ ^]^ In recent years, research on SEs in prostate cancer has gradually deepened, revealing their important roles in tumor initiation, progression and treatment. The AR, as the key driver in prostate cancer, can bind to SE regions to regulate the expression of downstream genes. The interaction between AR and SEs is of great significance for maintaining the proliferation and metastatic potential of cancer cells. FOXA1 is a pioneer factor that can reposition AR and other transcription factors to new super‐enhancers, altering gene expression patterns and promoting prostate cancer progression.^[^ ^22^ ^]^ Impeding SWI/SNF‐mediated enhancer accessibility represents a promising therapeutic approach for enhancer‐addicted CRPC.^[^ ^23^ ^]^

Ss are related to chemoresistance in various cancers, including small‐cell lung cancer, ovarian cancer, and breast cancer.^[^ ^24^, ^25^, ^26^ ^]^ Genes driven by SEs also reduce tumor cell sensitivity to chemotherapy. For instance, enzalutamide resistance emerges via SE‐driven CHPT1 overexpression in CRPC, regulated by AR binding to distinct enhancers and lncRNA‐mediated H3K27ac reader BRD4 interaction, highlighting SE‐targeted CHPT1 inhibition as a therapeutic strategy to overcome enzalutamide resistance.^[^ ^27^ ^]^ Additionally, mounting evidence suggests that inhibiting SE‐driven genes is a viable anti‐cancer strategy. For example, the small‐molecule inhibitor ML264, which targets KLF5, mitigates colorectal cancer progression both in vivo and in vitro.^[^ ^28^ ^]^ In liposarcoma, the bromodomain (BET) protein inhibitor ARV‐825 can inhibit the transcriptional regulatory loop composed of FOSL2, MYC and RUNX1—which holds regulatory dominance in liposarcoma and overcomes the resistance of liposarcoma to the clinical chemotherapeutic drug trabectedin.^[^ ^29^ ^]^

This study performs ChIP‐Seq targeting H3K27ac on CRPC cell line C42B and its Abiraterone‐resistant counterpart C42B‐ABi, identifying resistance‐specific SE‐associated genes. The analysis revealed a transcriptional regulatory loop driven by super‐enhancers in C42B‐ABi, comprising BCL6, NFIB and SMAD3, which are highly expressed in C42B‐ABi cells. Our research indicates that the SE‐driven transcriptional regulatory loop composed of BCL6, NFIB and SMAD3 is instrumental in sustaining CRPC resistance to Abiraterone. These three transcription factors directly co‐bind to each promoter and enhancer, thereby activating mutual transcription and collaboratively remodeling the transcriptome of resistant CRPC cells. Mechanistically, this loop promotes resistance through two distinct pathways: 1) regulating the cell cycle to facilitate resistance; 2) activating cholesterol synthesis to promote the synthesis of testosterone and dihydrotestosterone, which sustains resistance. This study delves into the transcriptional aberrations occurring during the development of Abiraterone resistance in CRPC and posits that targeting this loop and its downstream targets presents a potential therapeutic strategy to overcome Abiraterone resistance in CRPC.

## 2 Results

### 2.1 Identification of the C42B‐ABi‐Specific SE‐Driven TF Loop

**Figure 1:**
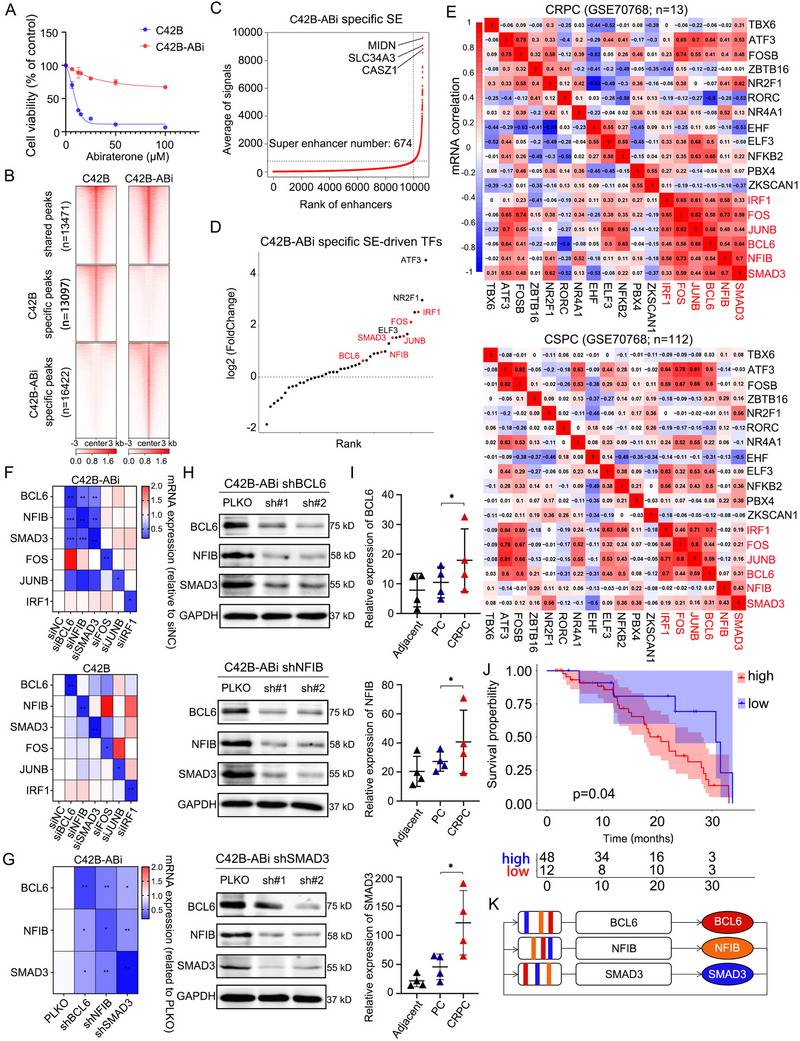
Screening of SE‐driven TF circuit specific to C42‐ABi cells. A) Treatment of C42B and C42B‐ABi cells with abiraterone, MTS assay performed to measure cell viability after 72 h. B) In C42B and C42B‐ABi cells, employing H3K27ac antibody for ChIP‐Seq to identify specific and shared peaks within both cell lines. C) Conducting ROSE analysis using the 16422 C42B‐Abi‐specific peaks to identify super enhancers. D) The volcano plot illustrates the differential expression of the 44 transcription factors, which are part of the 674 super enhancers, between C42B‐ABi and C42B cells. E) The correlation analysis of the expression of 18 transcription factors in tissues from CRPC and CSPC patients. F,G) Expression heatmap. Using siRNA (F) or shRNA (G) to knock down one of the genes, the mRNA levels of the other indicated genes were measured. Statistical significance was determined using Two‐way ANOVA Dunnett's multiple comparisons test. n = 3 independent experiments. *, P < 0.05; **, P < 0.01;***, P < 0.001. H) Using shRNA knock down BCL6, SMAD3, or NFIB in C42B‐ABi, the protein expression of these three molecules was analyzed by Western blot. I, The mRNA expression of indicated genes in adjacent (n = 4), primary cancer (PC, n = 4), and castration‐resistant prostate cancer (CRPC, n = 4) groups. Two‐way ANOVA Dunnett's multiple comparisons test, n = 3, *, P < 0.05. J, Kaplan‐Meier analyses was conducted by categorizing the cases into BCL6, SMAD3, and NFIB simultaneously high expression and low expression group. K, Diagram of the SE‐driven TF circuit.

In our previous work, we established the ABi‐resistant cell line C42B‐ABi that is significantly tolerated with Abiraterone treatment relative to the parent cell line C42B, the IC_50_ are over 100 and 9.3 µm respectively (**Figure** **1**A). To rigorously validate the authenticity of C42B‐ABi, we conducted systematic comparative analyses. The results demonstrated differential abiraterone sensitivity between parental C42B and resistant C42B‐ABi cells using both in vitro culture systems and subcutaneous xenograft models in mice (Figure S1A–F, Supporting Information). Systematically compared RNA‐Seq and whole‐exome sequencing (WES) data from these cell lines with published studies through comprehensive bioinformatic analysis (Figure S1G–J, Supporting Information), confirming C42B‐ABi's resistance‐associated signatures. To identify C42B‐ABi specific SE related peaks, we performed ChIP‐seq targeting H3K27ac in C42B and C42B‐ABi cell lines, the result shows that two cell lines shared 13471 peaks (31.33%), 13097 peaks are C42B specific (30.47%), and 16422 peaks are C42B‐ABi specific (38.2%) (Figure 1B). This indicated a different landscape of chromatin opening between C42B and C42B‐ABi cells. Interestingly, 674 C42B‐ABi specific SEs were identified from 16422 C42B‐ABi specific peaks (Figure 1C; Table S3, Supporting Information), while 403 SEs identified from 13097 C42B specific peaks (Figure S2A, Supporting Information). There are 247 C42B‐ABi‐specific SE‐driven genes up‐regulated in C42B‐ABi cells. HALLMARK analysis shows that these genes are significantly enriched with signaling, survival and response to hormone pathways (Figure S2B, Supporting Information), which are seen to be tightly involved in C42B resistant to Abiraterone. Furthermore, we found 18 of C42B‐ABi specific SE driven genes are transcription factors and up‐regulated in C42B‐ABi cells (Figure 1D; Table S3, Supporting Information), which could be highly correlated with malignant progression of prostate cancer, such as ELF3, reported to orchestrate a positive feedback loop with ESE1 activates NF‐κB and drives prostate cancer progression.^[^ ^30^ ^]^ We further note that the top‐ranked genes ATF3 and NR2F1 have been implicated in prostate cancer. ATF3 directly inhibits AR transcriptional activity and downstream targets (e.g., PSA), while NR2F1 loss promotes tumor heterogeneity and enzalutamide resistance. However, their roles in abiraterone‐resistant CRPC remain unexplored. To unveil whether there is a transcriptional regulatory loop in Abiraterone‐resistant CRPC, we analyzed the RNA‐seq data of tumor tissues in 13 CRPC patients to conduct a Pearson correlation analysis of the 18 transcript factors. Our results suggest the potential existence of a transcriptional regulatory loop, composed of IRF1, FOS, JUNB, BCL6, SMAD3 and NFIB. In contrast, there is no correlation in the expression of the six transcription factors within 112 PC tumor tissues (Figure 1E).

To verify the specificity of this transcriptional loop, we performed ChIP‐seq targeting H3K27ac in five cases of CRPC tumor tissues and corresponding para‐cancer tissue, and three cases of CSPC tumor tissues. ChIP‐seq data from 15 cases of CRPC PDX model tumor tissues, 24 cases of CSPC tumor tissues, and 28 cases of para‐cancer tumor tissues targeted H3K27ac in the GEO database were integrated.^[^ ^31^ ^]^ The result showed that the signal of H3K27ac of the 6 transcript factors in the enhancer region of the C42B‐ABi cell line or CRPC or CRPC‐PDX groups were higher than relative CSPC or adjacent groups (Figure S2C, Supporting Information). To validate this regulatory circuit, we attenuated the expression of the six transcription factors individually using siRNA and measured the expression of the remaining transcript factors in C42B‐ABi and C42B cell lines. The mutual regulation is only present among BCL6, NFIB and SMAD3 in C42B‐ABi cells and does not exist in C42B cells (Figure 1F, Figure S2D, Supporting Information). The validation results of shRNA are consistent with those of siRNA in C42B‐ABi (Figure 1G). Moreover, knocking down either BCL6, NFIB or SMAD3 results in a decrease in the protein levels of these three proteins in C42B‐ABi cells (Figure 1H). Conversely, over‐expressing each of the three proteins individually leads to an increase in the three proteins (Figure S2E, Supporting Information).

Importantly, we investigated the differential expression of BCL6, NFIB and SMAD3 in the tumor tissue samples and cell lines, with the findings indicating that these three genes are most highly expressed in CRPC and C42B‐ABi cells (Figure 1I, Figure S2F,G, Supporting Information). And the correlation between BCL6, NFIB and SMAD3 is only present in CRPC tumor tissues and not in CSPC tumor tissues or other adenocarcinoma tissues (e.g., esophageal cancer and breast cancer) (Figure S2H, Supporting Information). This indicates that the transcriptional loop composed of BCL6, NFIB and SMAD3 has better specificity and correlation in CRPC. Furthermore, we examined the correlation between the expression of BCL6, NFIB and SMAD3 and patient survival by proccessing the data of the public article.^[^ ^32^ ^]^ The prognosis of CRPC patients is poorer when all three genes are simultaneously highly expressed than when they are expressed at a low level (Figure 1J), and also poorer than when BCL6 or SMAD3 is individually highly expressed (Figure S2I, Supporting Information). These results demonstrate that BCL6, NFIB and SMAD3 form a specific transcriptional regulatory circuit in C42B‐ABi cell line (Figure 1K).

### 2.2 A Direct Transcriptional Activation Effect among BCL6, SMAD3 and NFIB

**Figure 2:**
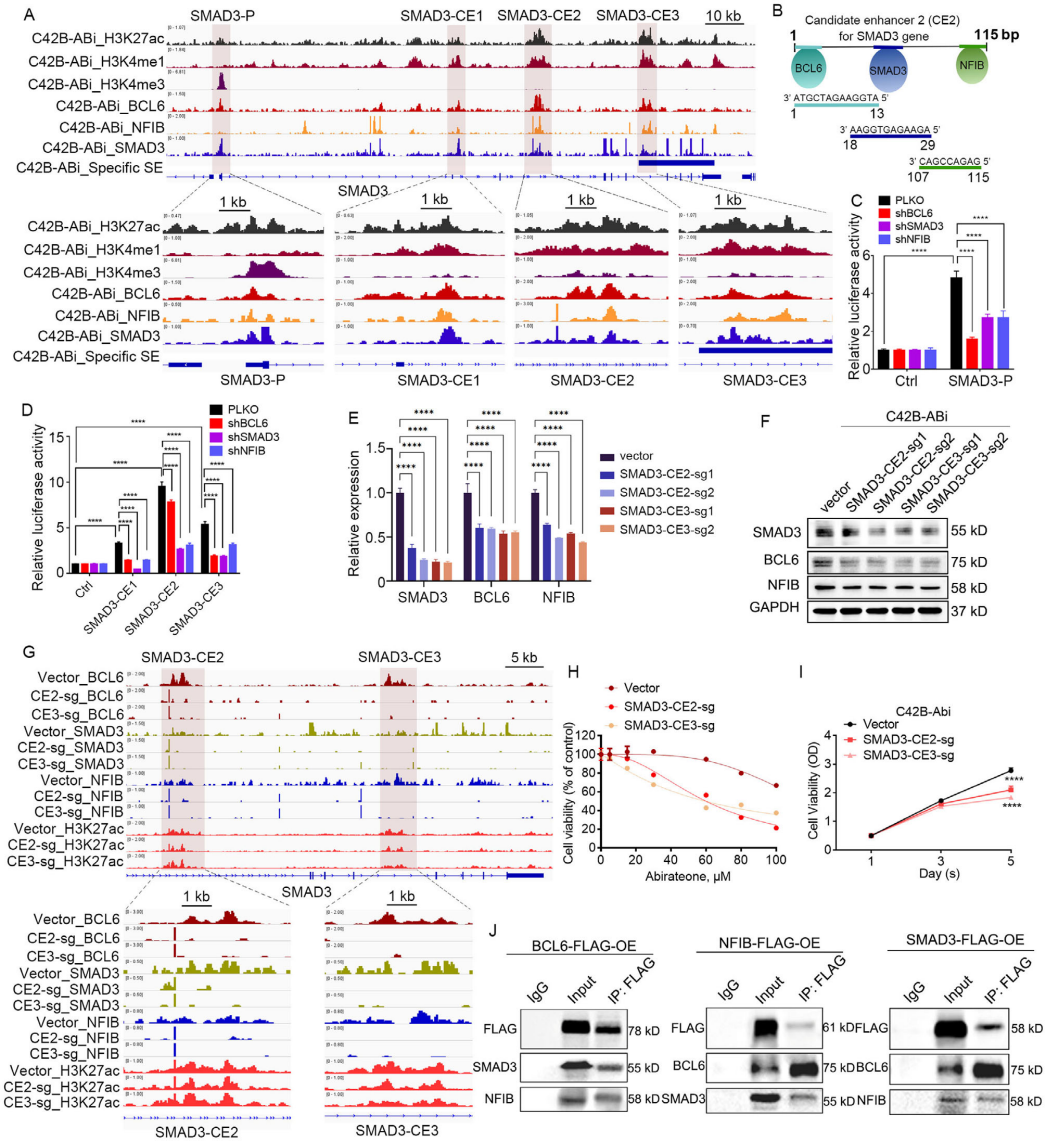
Validating the mechanism of the SE‐driven TF circuit. A) Integrative Genomics Viewer (IGV) plots of CUT&Tag showing co‐occupancy of BCL6, SMAD3, and NFIB at the promoter and enhancer of the SMAD3 gene locus. B) A schematic representation of the nearest distance pattern of BCL6, NFIB, ans SMAD3 motifs at SMAD3‐CE2. C, D, Promoter (C) and enhancer (D) activities were measured by luciferase reporter assays in C42B‐ABi cells in either the presence or absence of knockdown indicated TF. Two‐way ANOVA Dunnett's multiple comparisons test. n = 3. ****, P < 0.0001. E) The mRNA expression of BCL6, SMAD3, and NFIB were measured on C42B‐ABi cells transfected either vector or sgRNA targeting SMAD3‐CE2. Two‐way ANOVA Dunnett's multiple comparisons test. n = 3. ****, P < 0.0001. F) The protein expression of BCL6, SMAD3, and NFIB were measured on C42B‐ABi cells transfected either vector or sgRNA targeting SMAD3‐CE2. G) C42B‐ABi cells transfected either vector or sgRNA targeting SMAD3‐CE2 and SMAD3‐CE3, integrative Genomics Viewer (IGV) plots of CUT&Tag showing co‐occupancy of BCL6, SMAD3, and NFIB at the enhancers of the SMAD3 gene locus. H) Abiraterone dose‐response curves for C42B‐ABi cells transfected either vector or sgRNA targeting SMAD3‐CE2 and SMAD3‐CE3. I) C42B‐ABi cells transfected either vector or sgRNA targeting SMAD3‐CE2 and SMAD3‐CE3, cell proliferation were detected. One‐way ANOVA Dunnett's multiple comparisons test. n = 3. ****, P < 0.0001. J) Overexpress Flag‐tagged BCL6, SMAD3, and NFIB in C42B‐ABi cells individually, perform co‐IP experiments using a Flag antibody, and detect BCL6, SMAD3, and NFIB via Western Blot. IgG serves as the negative control.

To ascertain the regulatory mechanism between BCL6, SMAD3 and NFIB, we conducted CUT&Tag using specific antibodies individually targeting the three transcription factors and the epigenetic modification marker proteins H3K27ac, H3K4me1 and H3K4me3. The results demonstrate that BCL6, SMAD and NFIB collectively bind to each other's promoters and candidate enhancers (**Figure 2**A; Figure S3A,B, Supporting Information). Importantly, the maximum distance between the motifs of the three transcription factors in co‐binding region is 115 bp (SAMD3‐E1), and the minimum is only 23 bp (NFIB‐E1), supporting that the three transcription factors collectively bind to the same chromosomal location (Figure 2B; Figure S3C, Supporting Information). Notably, we also compared the binding differences of the three transcription factors at representative enhancer regions between two cell lines using comparative ChIP‐PCR analysis. In C42B‐ABi cells, this regulatory loop exhibits higher enrichment at the SMAD3‐CE2/CE3, BCL6‐CE3, and NFIB‐CE1 loci compared to C42B cells. Specifically, in C42B cells, NFIB and SMAD3 exhibit no enrichment at these regions, while BCL6 shows partial enrichment (Figure S3D–G, Supporting Information). These findings provide critical evidence demonstrating the specificity relevance of this regulatory circuit in C42B‐ABi cells.

To validate the co‐binding of three transcription factors at the promoter and enhancer regions at the SMAD3 gene locus. We identified a promoter where three transcription factors co‐bind, named SMAD3‐P, along with three co‐binding candidate enhancers designated as SMAD3‐CE1, SMAD3‐CE2, and SMAD3‐CE3 (Figure 2A). These sequences were individually incorporated into the luciferase reporter vector and transfected into C42B‐ABi cells. The findings revealed a substantial increase in luciferase activity for SMAD3‐P, SMAD3‐CE1, SMAD3‐CE2, and SMAD3‐CE3 compared to the control, while knockdown of BCL6, SMAD3 and NFIB resulted in a significant decrease in luciferase activity (Figure 2C,D). Furthermore, targeted sgRNA sequences were designed against the SMAD3‐CE2 and CE3, the sequence with the higher activity, and integrated into the pLentiCRISPR V2 vector. The result indicated that the destruction of SMAD3‐CE2 and CE3 can not only suppress the expression of mRNA and protein levels of SMAD3 but also BCL6 and NFIB (Figure 2E,F). In conclusion, BCL6, SMAD3 and NFIB activate their own as well as each other's transcriptional expression by directly binding at mutual promoters and enhancers. To investigate the roles of these two enhancers in mediating the regulatory loop's control over SMAD3. We performed CUT&Tag experiments using antibodies against BCL6, NFIB, SMAD3, and H3K27ac in the control group (Vector) and SMAD3‐CE2/CE3 mutant groups (CE2‐sg and CE3‐sg). The results are shown in Figure 2G. The binding of all three transcription factors (BCL6, NFIB, and SMAD3) at the SMAD3‐CE2 and CE3 loci was significantly reduced compared to the control group. Concurrently, H3K27ac signals (marking chromatin openness) at these loci also showed varying degrees of attenuation following SMAD3‐CE2/CE3 mutations. These results indicate that mutations in the CE2/CE3 regions disrupt the co‐binding of the three transcription factors and alter chromatin accessibility at these enhancer loci. This finding is critical for demonstrating enhancer‐mediated regulation of SMAD3 by the three transcription factors. Furthermore, functional assays revealed that SMAD3‐CE2/CE3 mutations led to increased sensitivity to abiraterone (Figure 2H) and impaired cell proliferation (Figure 2I). These phenotypic changes mimic the effects of SMAD3 knockdown, suggesting that CE2/CE3 mutations functionally inactivate SMAD3‐mediated regulatory activity. Due to the co‐binding of three transcription factors at a shared enhancer or promoter elements and their concerted activation of transcription, we speculate a potential existence of protein‐level interaction among them. Co‐IP assays were performed to verify this, and Figure 2G illustrates that BCL6, SMAD3 and NFIB exhibit mutual protein‐level interactions. These results indicate that BCL6, SAMD3, and NFIB compose stable complexes in Abiraterone‐resistant CRPC.

### 2.3 BCL6, SMAD3, and NFIB Cooperatively Orchestrate the Transcriptome of C42B‐ABi Cells

**Figure 3:**
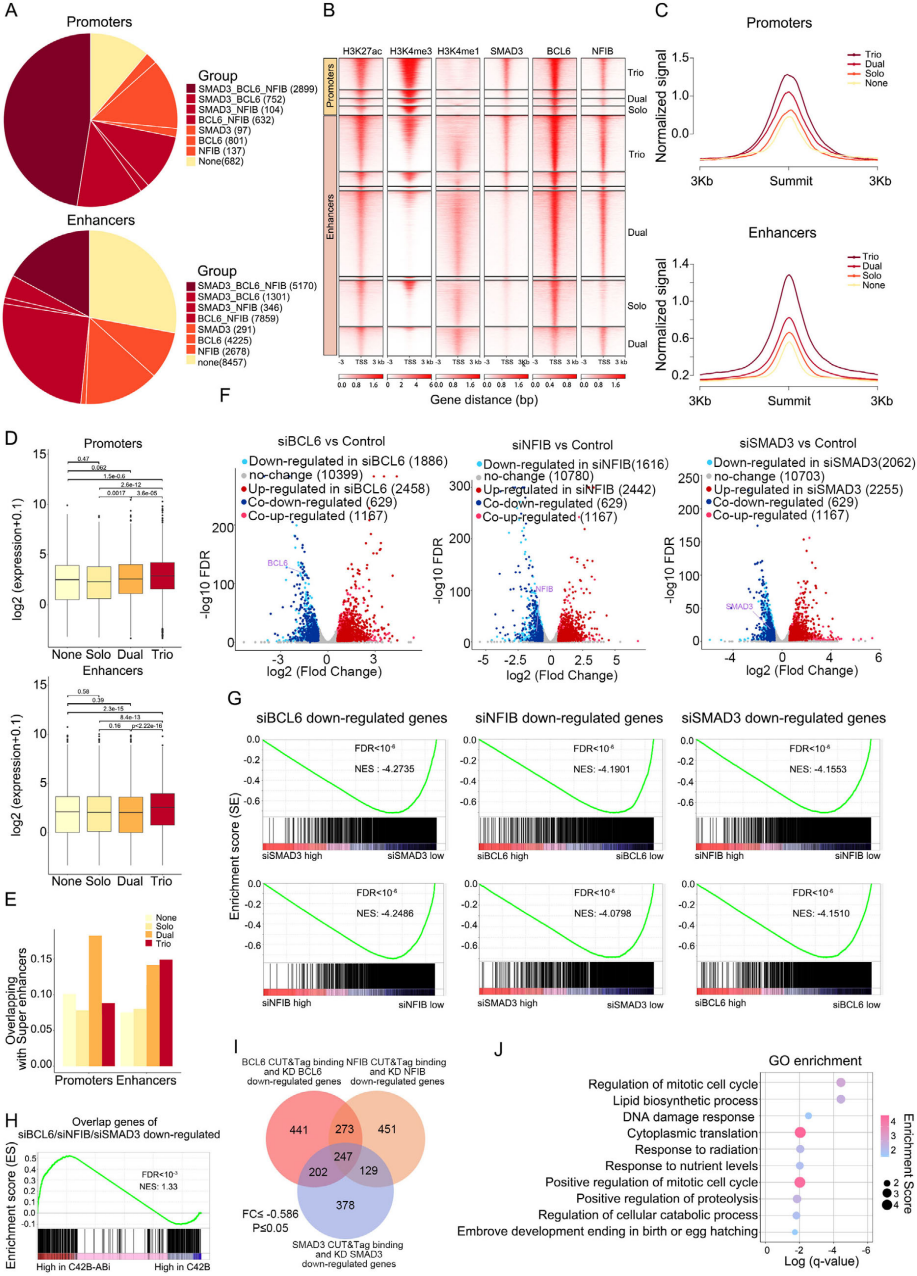
Transcriptional cooperativity between BCL6, SMAD3, and NFIB. A) The pie chart show the co‐binding overview of BCL6, SMAD3, and NFIB at promoters and enhancers. B) Heatmaps of CUT&Tag signals of indicated factors in C42B‐Abi cells, stratified by different combinatorial binding patterns. C) The signal intensity of H3K27ac among the peaks of indicated groups. D) mRNA expression of target genes regulated by the peaks of indicated groups in C42B‐ABi cells. E) The overlapping of indicated groups of peaks with super‐enhancers in C42B‐ABi cells. F) Volcano plots of RNA‐Seq after individually knocking down BCL6, SMAD3, and NFIB in C42B‐ABi cells. Up regulated in siTFs (BCL6, NFIB, SMAD3), log_2_ fold‐change>0.584, p‐value < 0.05; down regulated in siTFs (BCL6, NFIB, SMAD3), log_2_ fold‐change < ‐0.584 and p‐value < 0.05. G) Genes down‐regulated by knocking down BCL6 are subjected to GSEA enrichment analysis with the RNA‐Seq data of SMAD3 or NFIB knockdown. Similarly, down‐regulated genes from the SMAD3 or NFIB knockdown are analyzed in the same manner. H) The overlap genes down‐regulated by knocking down BCL6, NFIB, and SMAD3 are subjected to GSEA enrichment analysis with the RNA‐Seq data of C42B‐ABi and C42B. I) Venn diagram of down‐regulated genes following silencing of each single TFs and binding by them. J) Gene Ontology pathway enrichment analysis of overlapping genes.

To unravel the transcriptional regulatory mechanisms of BCL6, SMAD3 and NFIB in Abiraterone‐resistant CRPC, we analyzed the epigenomic characteristics of their occupancy in C42B‐ABi cells. Based on the co‐occupancy of H3K27ac and H3K4me3, 6104 promoters were identified, while 30 327 enhancers were identified by H3K27ac and H3K4me1 co‐occupying. The quantity of enhancers far exceeds that of promoters. The number of concurrent bindings of BCL6, SMAD3 and NFIB either in promoter or enhancer is classified into four groups: trio, dual, solo, and none. Notably, 71.9% of promoters and 48.4% of enhancers are co‐binding by at least two factors individually (**Figure** **3**A,B; Table S3, Supporting Information), indicating a profound synergistic occupancy among the three transcription factors. The intensity of H3K27ac indicates that chromatin accessibility is highest in the trio group for both promoters and enhancers (Figure 3C). Correspondingly, the expression of target genes is also highest in the trio cohort compared with the other three (Figure 3D). Moreover, the enhancers occupied by the trio group demonstrate a higher proportion of overlap with super‐enhancers (Figure 3E). All of the results indicated the transcription factors BCL6, SMAD3 and NFIB synergistically activate the transcription of target genes in C42B‐ABi cells.

To further substantiate the synergistic regulation of transcripts by the three transcription factors. We employed siRNA‐mediated knockdown of BCL6, SMAD3 and NFIB for RNA‐Seq analysis in C42B‐ABi cells (Figure 3F; Table S3, Supporting Information). Interestingly, Gene Set Enrichment Analysis (GSEA) showed genes that were down‐regulated by silencing of BCL6 were significantly enriched in genes that were also down‐regulated following silencing of the other two factors (NFIB or SMAD3) (Figure 3G). The same phenomenon was also observed in NFIB and SAMD3, suggesting that the three transcription factors exhibit remarkable similarity in orchestrating transcripts in C42B‐ABi cells. Correspondingly, the up‐regulated genes also show significant similarity (Figure S4A, Supporting Information). Moreover, the genes that are commonly down‐regulated after silencing the three transcription factors are significantly enriched in the gene set characterized by high expression of C42B‐ABi (Figure 3H). These results suggest that BCL6, SAMD3 and NFIB collaboratively remodel transcription to facilitate ABi‐resistant in CRPC.

Additionally, we investigated whether there is a regulatory relationship between this transcriptional regulatory circuit and AR, the driver gene of prostate cancer. First, we knocked down AR and found that the mRNA and protein levels of the three transcription factors were upregulated, while down‐regulated with AR over expression (Figure S4B–D, Supporting Information). We compared the expression levels of AR and its target protein PSA between C42B and C42B‐ABi cells and found their protein level significantly decreased in the Abiraterone‐resistant cells (Figure S4E, Supporting Information), which is a consensus about the treatment of androgen receptor signaling inhibitors has led to an increase of CRPC de‐differentiate into AR‐negative disease.^[^ ^4^, ^6^, ^33^ ^]^ And silencing BCL6, SMAD3 and NFIB, the protein levels of AR were up‐regulated (Figure S4F, Supporting Information), suggesting that there is an inverse regulatory relationship between AR and this transcriptional regulatory circuit. And these results demonstrated that inhibiting the transcriptional loop could recover the sensitivity of Abiraterone via increasing the expression of AR in C42B‐ABi cells to some extent.

### 2.4 The factors of the SE‐Driven TF Loop Promote the Malignant Phenotype of ABi‐Resistant CRPC

To investigate the biological processes that the transcriptional regulatory network promotes in conferring drug resistance, we employed CUT&Tag and RNA‐Seq data for BCL6, SMAD3 and NFIB, and found 247 genes, representing downstream target genes jointly transcriptional regulated by BCL6, SMAD3 and NFIB (Figure 3I; Table S3, Supporting Information). The top two enriched pathways are the “Lipid Biosynthetic Process” and “Regulation of Mitotic Cell Cycle” according to Gene Ontology pathway enrichment analysis of the 247 genes (Figure 3J), suggesting a promotion of proliferation with the loop‐mediated facilitation of Abiraterone resistance in CRPC.

**Figure 4:**
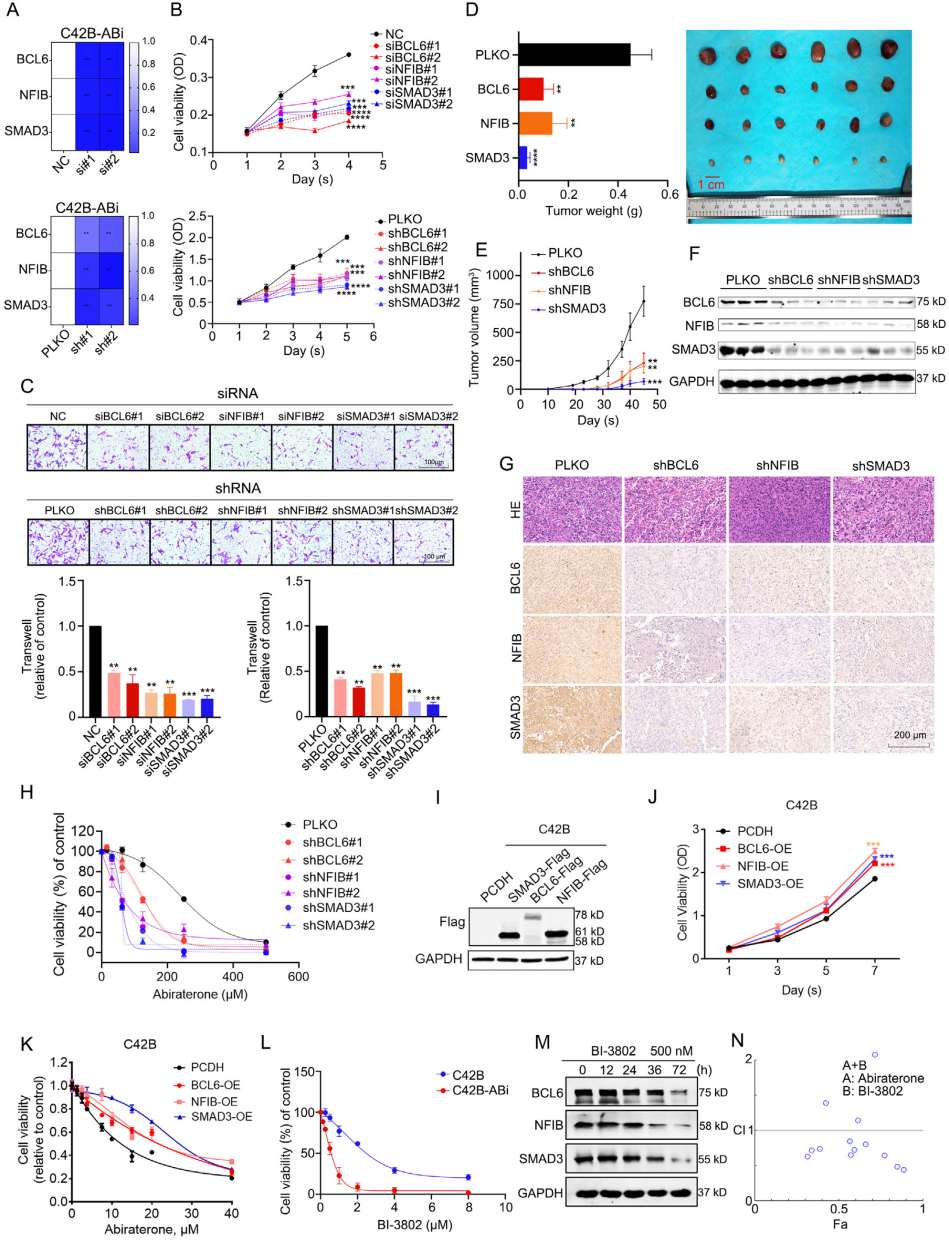
BCL6, SMAD3, or NFIB loss inhibits cell proliferation and enhances sensitivity to abiraterone in C42B‐Abi cells. A) The knockdown efficiency of siRNA and shRNA. One‐way ANOVA Dunnett's multiple comparisons test. n = 3. **, P < 0.01. B,C), Silencing BCL6, SMAD3, and NFIB individually by siRNA or shRNA, cell proliferation (B) and cell migration (C) were detected. One‐way ANOVA Dunnett's multiple comparisons test. n = 3. **, P<0.01; ***, P < 0.001; ****, P < 0.0001. D) Inoculate castrated nude mice with C42B‐ABi cells that have knocked down BCL6, SMAD3, or NFIB. At the terminal of the experiments, the mice were sacrificed, and then the tumors were excised and weighed. One‐way ANOVA Dunnett's multiple comparisons test. n = 6. **, P < 0.01; ****, P < 0.0001. E) The tumor sizes were recorded every day, starting from the tenth day post‐inoculation. One‐way ANOVA Dunnett's multiple comparisons test. n = 3. **, P < 0.01; ***, P < 0.001. F) The indicated proteins in tumor tissue were detected by Western blots. G) Immunohistochemical (IHC) analysis of the indicated proteins in tumor tissue. H) Abiraterone dose‐response curves for C42B‐ABi cells with BCL6, SMAD3, or NFIB knockdown. I) Overexpress Flag‐tagged BCL6, SMAD3, and NFIB in C42B cells individually and detect the protein level of Flag via Western Blot assay. J) Overexpress BCL6, SMAD3, and NFIB in C42B cells individually, cell proliferation were detected. One‐way ANOVA Dunnett's multiple comparisons test. n = 3. ***, P < 0.001. K) Abiraterone dose‐response curves for C42B cells with BCL6, SMAD3, or NFIB over‐expression. L) BI‐3802 dose‐response curves for C42B and C42B‐ABi cells after 3 days. M) C42B‐ABi cells were treated with BI‐3802, and then the indicated proteins were detected by Western blots. N) The CI value of BI‐3802 combining with abiraterone were smaller than one, which indicated synergy effect on BI‐3802 with abiraterone. CI = 1 means additive effect, CI<1 means synergism, and CI>1 means antagonism. Fraction affected (Fa) is the proportion of cells affected by combination treatment.

Individually silencing BCL6, SMAD3 and NFIB using siRNA or shRNA resulted in inhibited proliferation and migration capabilities of the C42B‐ABi cells (**Figure** **4**A–C). Furthermore, following castration surgery in nude mice, subcutaneous implantation of C42B‐ABi cells with stable knockdown of BCL6, SMAD3 and NFIB significantly suppressed tumor proliferation compared to the control group (Figure 4D,E). Tumor tissues were isolated for Western blot and Immunohistochemistry analysis, revealing a protein‐level inter‐regulatory relationship among the three factors (Figure 4F,G). We also observed a negative regulatory relationship between this loop and AR/PSA in subcutaneous tumor tissues (Figure S4G,H, Supporting Information). The findings indicate that the SE‐driven TF loop composed of BCL6, SMAD3 and NFIB are promoting the malignant progression of Abiraterone‐resistant CRPC.

**Figure 5:**
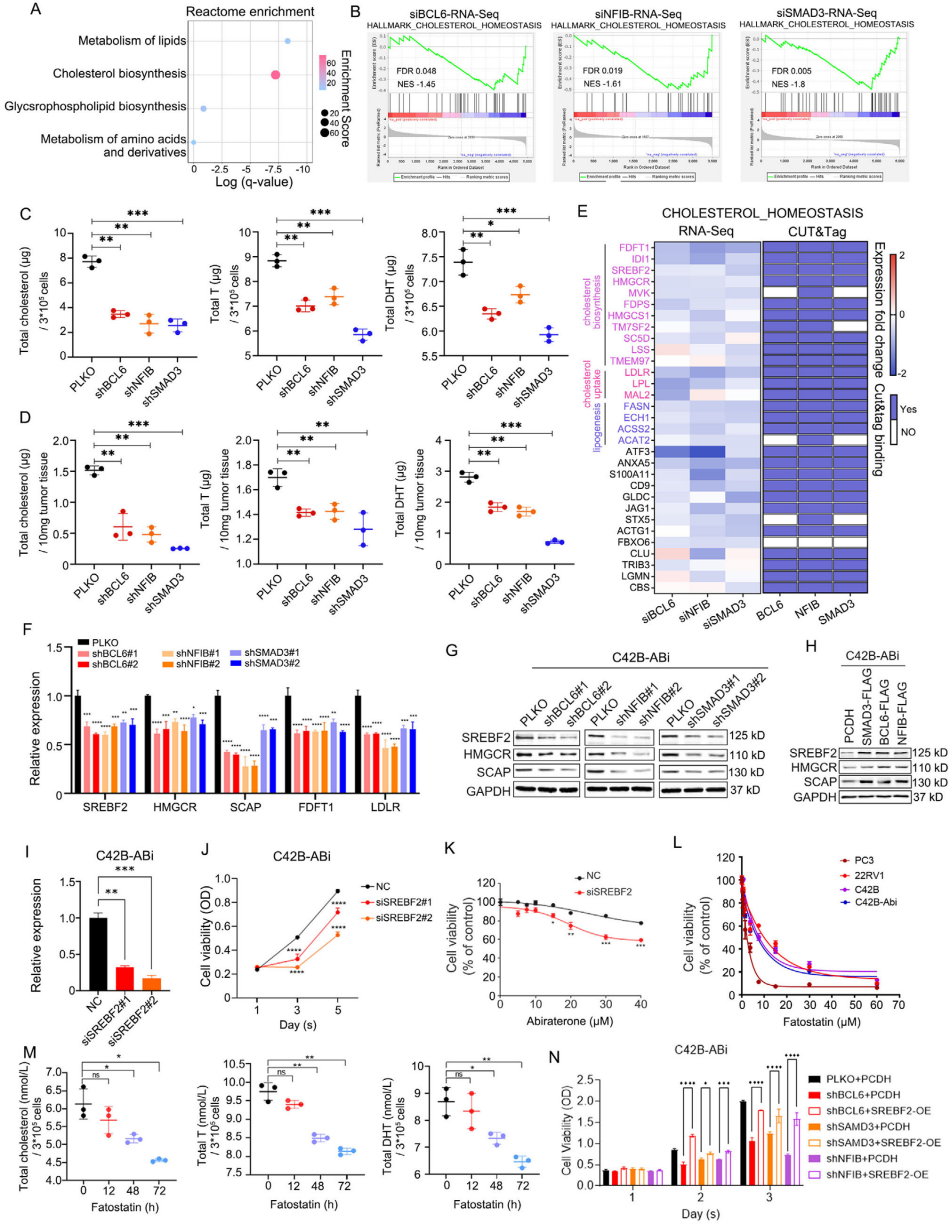
The super‐enhancer‐driven transcription factor loop activates the cholesterol biosynthesis. A) Reactome enrichment analysis of the genes enriched in the“Lipid biosynthetic process”of Figure 3J. B) the RNA‐Seq data of BCL6, SMAD3, and NFIB knockdown are subjected to GSEA enrichment analysis with the gene sets associated with cholesterol homeostasis. C,D) Intracellular cholesterol, testosterone (T), and dihydrotestosterone (DHT) levels measured following the individual knockdown of BCL6, SMAD3, and NFIB in C42B‐ABi cells (C) and tumor xenografts in mice (D). One‐way ANOVA Dunnett's multiple comparisons test. n = 3. *, P <0.05; **, P < 0.01; ***, P < 0.001. E) Heatmaps illustrating the mRNA expression and binding patterns of the gene collection enriched in cholesterol homeostasis. F,G) The mRNA (F) and protein (G) expression level of indicated genes were detected in C42B‐ABi cells following silencing of BCL6, SMAD3, and NFIB. Two‐way ANOVA Dunnett's multiple comparisons test. n = 3. *, P <0.05; **, P < 0.01; ***, P < 0.001; ****, P < 0.0001. H) The protein expression of SREBF2, HMGCR, and SCAP were measured following the over‐expression of BCL6, SMAD3, or NFIB in C42B‐ABi cells. I,J) Silencing SREBF2 by siRNA in C42B‐ABi cells (I), the cell proliferation (J) was detected. One‐way ANOVA Dunnett's multiple comparisons test. n = 3. **, P < 0.01; ***, P < 0.001; ****, P < 0.0001. K) Abiraterone dose‐response curves for C42B‐ABi cells after 3 days of SREBF2 knockdown. One‐way ANOVA Dunnett's multiple comparisons test. n = 3. *, P <0.05; **, P < 0.01; ***, P < 0.001. L) In vitro MTS proliferation assay of prostate cancer cells in the presence of different doses of fatostatin (SREBF2 inhibitor). M) Intracellular cholesterol, testosterone (T), and dihydrotestosterone (DHT) levels measured with fatostatin treatment in C42B‐ABi cells. One‐way ANOVA Dunnett's multiple comparisons test. n = 3. *, P <0.05; **, P < 0.01. N) Overexpress SREBF2 after silencing BCL6, SMAD3, and NFIB by shRNA in C42B‐ABi cells, the cell proliferation was detected. Two‐way ANOVA Dunnett's multiple comparisons test. n = 3. *, P <0.05; ***, P < 0.001; ****, P < 0.0001.

Subsequently, we investigated the regulatory roles of the three transcription factors on Abiraterone resistance. The sensitivity of C42B‐ABi to Abiraterone was heightened upon individual knockdown of BCL6, SMAD3 and NFIB (Figure 4H). Corresponding, over‐expression of BCL6, SAMD3 and NFIB in the C42B can promote cell proliferation, inhibit the cell migration and the sensitivity to Abiraterone (Figure 4I–K; Figure S4I, Supporting Information). BI‐3802, a BCL6 inhibitor, significantly decreases the cell viability of C42B‐ABi and C42B. Due to higher BCL6 expression, compared to C42B, BI‐3802 exhibits a more notable inhibitory effect on C42B‐ABi (Figure 4L). Moreover, the protein levels of BCL6, SAMD3 and NFIB were suppressed with BI‐3802 treatment (Figure 4M). Notably, BI‐3802 can synergistically cooperate with Abiraterone to suppress the viability of C42B‐ABi cells (Figure 4N). Taken together, the transcription factor loop components driven by SE, namely BCL6, SMAD3 and NFIB, contribute to fortifying the resistance of C42B‐ABi against Abiraterone. Conversely, targeted suppression of this loop could increase the sensitivity of C42B‐ABi to Abiraterone.

### 2.5 The SE‐Driven TF Loop Activates Cholesterol Homeostasis

We have demonstrated the role of the transcriptional regulatory network in driving Abiraterone resistance in C42B‐ABi cells. However, the specific mechanism through which it operates remains unknown. The pathway enrichment analysis suggests that BCL6, SMAD3 and NFIB collaboratively regulate the pathway of lipid biosynthetic processes (Figure 3I). To validate this, we examined the lipid droplet abundance within cells following the knockdown of BCL6, SMAD3 and NFIB individually, revealing a significant decrease in lipid droplets (Figure S5A,B, Supporting Information). The genes enriched in the “lipid biosynthetic processes” were further subjected to Reactome enrichment analysis, and the results indicated a significant enrichment in the “cholesterol biosynthesis pathway” (**Figure** **5**A). HALLMARK analysis revealed that the overlap genes down‐regulated after the knock‐down of three transcription factors are significantly enriched in cholesterol homeostasis (Figure 5B). Genes associated with cholesterol homeostasis are significantly enriched in the gene set with high expression in C42B‐ABi compared to C42B cells (Figure S5C, Supporting Information). Cholesterol is an important component of cell membranes, and it acts as a precursor to bile acids, steroid hormones, and 7‐dehydrocholesterol to regulate cell proliferation and survival. Several studies have shown that abnormal cholesterol homeostasis, including the dynamic processes of uptake, efflux, synthesis, and degradation, can promote the malignant progression of tumors.^[^ ^34^ ^]^ Our findings suggest that the SE‐driven TF loop may regulate cholesterol homeostasis to sustain the resistance of Abiraterone. To validate this, we quantified the intracellular levels of cholesterol and its synthetic products testosterone and dihydrotestosterone, which activate the androgen receptor (AR) signaling pathway. The results indicate a significant decrease in cholesterol, testosterone, and dihydrotestosterone following the individual knockdown of BCL6, SMAD3 and NFIB (Figure 5C). A similar phenomenon was observed in tumor xenografts in mice; however, not in the serum of the mice (Figure 5D; Figure S5D, Supporting Information). Elevated levels of cholesterol, testosterone, and dihydrotestosterone were observed upon individual overexpression of each TF in C42B cells, and all three biomarkers were significantly enrichment in C42B‐ABi cells (Figure S5E,F, Supporting Information). These results illustrated that the SE‐driven TF loop promotes an increase in intratumoral cholesterol, testosterone, and dihydrotestosterone.

To elucidate the specific mechanism by which the SE‐driven TF loop regulates cholesterol homeostasis, we compiled genes enriched in cholesterol homeostasis and discovered these genes involved in cholesterol synthesis, cholesterol uptake, and lipogenesis, with genes related to cholesterol synthesis being the most prevalent (Figure 5E). With low cholesterol levels, SCAP binds to sterol regulatory element binding proteins (SREBP) and transports from the ER to the Golgi.^[^ ^35^ ^]^ The SREBP is then proteolytically cleaved and enters the nucleus to activate the transcription of target genes, including the core regulators of the mevalonate pathway HMGCR, FDFT1, and cholesterol uptake receptors LDLR.^[^ ^36^ ^]^ Thus, we validated a subset of genes associated with cholesterol synthesis and uptake. Our findings reveal a significant reduction in mRNA levels of these genes upon knockdown of BCL6, SMAD3 and NFIB (Figure 5F). Moreover, there is a substantial decrease observed in the protein abundance of SCAP, SREBF2 and HMGCR following the depletion of three transcription factors (Figure 5G). Conversely, SCAP, SREBF2 and HMGCR were up‐regulated when the three transcription factors were over‐expressed (Figure 5H). CUT&Tag data indicate that BCL6, SMAD3 and NFIB collectively bind to the promoter or enhancer elements of these target genes (Figure S5G, Supporting Information). These data demonstrate that BCL6, SMAD3 and NFIB transcriptionally activate genes associated with cholesterol synthesis directly, thereby promoting cholesterol biosynthesis.

To validate the regulation of cholesterol‐mediated SE‐driven TF loop on Abiraterone resistance in C42B‐ABi cells, we focused on the SREBF2, given its role as a transcription factor governing genes associated with cholesterol synthesis. First, in C42B‐ABi cells, employing siRNA to silence SREBF2 led to a notable attenuation in cell proliferation and heightened sensitivity to Abiraterone (Figure 5I–K). Fatostatin, the inhibitor of SREBF2, significantly decreased cell viability of C42B and C42B‐ABi cells (Figure 5L). As expected, with the treatment of fatostatin, there was a time‐dependent decrease of cholesterol, testosterone, and dihydrotestosterone in C42B‐ABi cells (Figure 5M). Moreover, the protein level of SREBF2 and its targets HMGCR, FDFT1, and SCAP also decreased with fatostatin treatment (Figure S5H, Supporting Information). Furthermore, upon individually silencing BCL6, SMAD3 and NFIB, overexpression of SREBF2 was demonstrated to ameliorate the cell proliferation suppression, as same as the down‐regulation of HMGCR and FDFT1, caused by the knockdown of these three transcription factors (Figure 5N; Figure S5I, Supporting Information). Meanwhile, Lovastatin, the inhibitor of HMGCR also inhibits cell viability and synergistically coordinates with Abiraterone in suppressing cell proliferation (Figure S5J,K, Supporting Information).

### 2.6 The SE‐Driven TF Loop Mediates Cell Cycle Arrest in C42B‐ABi Cells

**Figure 6:**
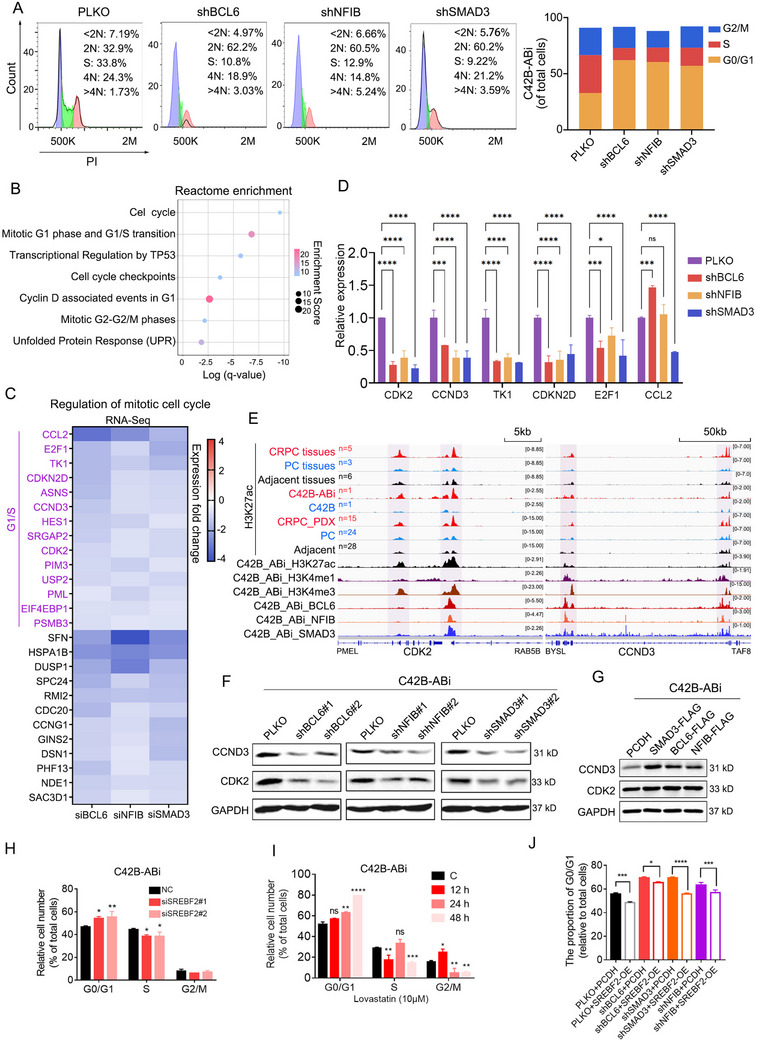
The super‐enhancer‐driven transcription factor loop mediates cell cycle arrest in the G0/G1 phase. A) Propidium iodide staining for cell cycle analysis. The bar chart represents the statistical data for each phase. B) Reactome enrichment analysis of the genes enriched in the“regulation of mitotic cell cycle”of Figure 3J. C) Heatmap showing the mRNA expression of the genes enriched in the “regulation of mitotic cell cycle”. D) The mRNA expression levels of CDK2 and CCND3 were assessed following the knockdown of BCL6, SMAD3, or NFIB in C42B‐ABi cells. E) IGV showing indicated protein binding at the CDK2 and CCND3 loci. F,G) The protein expression level of CDK2 and CCND3 were measured following the silence (F) or overexpress (G) of BCL6, SMAD3, or NFIB in C42B‐ABi cells. H) Silencing SREBF2 by siRNA in C42B‐ABi cells, the cell cycle was detected. One‐way ANOVA Dunnett's multiple comparisons test. n = 3. **, P < 0.01; ***, P < 0.001. I) C42B‐Abi cells were treated with 10 µm lovastatin (the inhibitor of HMGCR) for 12, 24, and 48 h, and the cell cycle was detected. One‐way ANOVA Dunnett's multiple comparisons test. n = 3. **, P < 0.01; ***, P < 0.001,****, P < 0.0001. J) Overexpress SREBF2 after silencing BCL6, SMAD3, and NFIB by shRNA in C42B‐ABi cells, the proportion of G0/G1 was detected. One‐way ANOVA Dunnett's multiple comparisons test. n = 3. *, P < 0.015, ***, P < 0.001,****, P < 0.0001.

We discovered that the SE‐driven TF loop promotes the progression of Abiraterone resistance by regulating the cell cycle (Figure 3I). To validate this, we individually silenced BCL6, SMAD3 and NFIB in C42B‐ABi cells, the cell cycle exhibited a notable blockade in the G0/G1 phase (**Figure** **6**A). Consistent with the experimental results, reactome analysis of genes clustered in “Regulation of Mitotic Cell Cycle” in figure 3I showed significant enrichment in the G1/S phase transition (Figure 6B). To reveal the mechanism, we examined the genes enriched in the “Regulation of Mitotic Cell Cycle” pathway of figure 3I according to RNA‐seq results (Figure 6C). By knocking down three transcription factors, we validated that the mRNA level of these genes was reduced (Figure 6D). We observed that the CDK2 and CCND3, which facilitate the transition of cells from the G1 phase to the S phase, are significantly down‐regulated after BCL6, SMAD3 and NFIB knockdown. Therefore, we hypothesize that silencing the loop may reduce the expression of CCND3 and CDK2 by regulating their transcription, resulting in the G0/G1 phase arrest in C42B‐ABi cells.

The CUT&Tag data demonstrate direct binding of the three transcription factors to the promoter and enhancer elements of the key regulatory genes CDK2 and CCND3 (Figure 6E). The protein expression of both CDK2 and CCND3 was inhibited following the individual knockdown of BCL6, SMAD3 and NFIB in C42B‐ABi cells (Figure 6F). Conversely, overexpression leads to an elevation in the protein expression of CDK2 and CCND3 (Figure 6G). In conclusion, these findings suggest that the transcriptional regulatory circuit directly activates CDK2 and CCND3 to regulate the cell cycle, thereby promoting the proliferation of Abiraterone‐resistant CRPC cells.

Interestingly, recent literature indicates that there is a tight crosstalk between the mevalonate pathway and the cell cycle.^[^ ^37^, ^38^ ^]^ Such as inhibition of HMGCR with high doses of statins primarily leads to cell cycle arrest at the G1 phase due to reduced availability of mevalonate‐derived non‐sterol isoprenoids. Furthermore, HMGCR inhibition can also block the cell cycle at G2/M because of the reduction of cholesterol availability. Inhibition of diphosphomevalonate decarboxylase (MVD) slows down S‐phase progression by affecting DNA replication. We speculate that cholesterol synthesis inhibition may lead to cell cycle arrest at the G0/G1 phase in C42B‐ABi cells. Therefore, we used siRNA and statins to respectively inhibit SREBF2 and HMGCR, and found the cell cycle was indeed arrested at the G0/G1 phase (Figure 6H,I). Furthermore, the over‐expression of SREBF2 can partially reverse the G0/G1 phase arrest induced by knockdown of the SE‐driven TF loop (Figure 6J). These results indicate that the SE‐driven TF loop can regulate the cell cycle by transcriptionally activating cell cycle‐related proteins, and by a bypass pathway, which is activates cholesterol metabolism.

## 3 Discussion

The transcriptional dysregulation mediated by super‐enhancers in prostate cancer has been reported. Such as targeting the SWI/SNF complex, which regulates abnormal chromatin remodeling, disrupting the transcriptional expression of super‐enhancer‐driven genes like AR, FOXA1, ERG and MYC, as well as their regulatory programs on downstream genes in prostate cancer.^[^ ^39^ ^]^ LSD1, BRD4 and FOXA1 form a transcriptional regulatory loop, binding to super‐enhancer elements to regulate the transcription of target genes. Inhibitors of LSD1 and BET exhibit potent synergistic effects, inducing tumor growth inhibition by disrupting the activity of multiple driver genes in CRPC.^[^ ^40^ ^]^ A set of aberrantly activated super‐enhancers identified in Enzalutamide‐resistant CRPC cells. The super‐enhancer driving oncogene CHPT1 catalyzes phosphatidylcholine synthesis to sustain Enzalutamide resistance.^[^ ^27^ ^]^ However, studies on super‐enhancer‐driven transcriptional dysregulation in Abiraterone‐resistant CRPC have not been reported.

This study has, for the first time, delineated the specific landscape of super‐enhancers in Abiraterone‐resistant CRPC cells by performing H3K27ac‐targeted ChIP‐seq on the C42B cell line and its Abiraterone‐resistant variant, C42B‐ABi cells. Genes associated with super‐enhancers exhibit higher expression levels than those regulated by typical enhancers, which is one of the key characteristics underlying the significant role of super‐enhancers. By integrating RNA‐Seq data from these two cell lines, we identified and validated a mutually regulatory loop driven by super‐enhancers. This loop, which is highly expressed in C42B‐ABi, is composed of BCL6, NFIB and SMAD3. Previous research indicates that SMAD3 is overexpressed in advanced prostate cancer tissues and promotes CRPC cell proliferation.^[^ ^41^ ^]^ NFIB facilitates tumor cell proliferation and resistance in hormone‐dependent breast cancer and colorectal cancer.^[^ ^42^ ^]^ NFIB is also identified to promote EMT and metastasis of CRPC.^[^ ^43^ ^]^ Initially identified as an oncogene in B‐cell lymphoma, BCL6 drives malignant phenotypes by inhibiting proliferation and DNA damage checkpoints, while obstructing terminal B‐cell differentiation. However, recent reports identified that BCL6 acts as a pro‐tumor growth factor in various malignancies, playing a crucial role in promoting resistance to anti‐tumor drugs and chemotherapy. Notably, no previous studies have reported the involvement of BCL6, NFIB and SMAD3 in Abiraterone‐resistant CRPC. In our study, we found they regulate each other's and their transcription by co‐binding to each other's and their own enhancer or promoter regions (Figure 2). Moreover, they collaboratively reshape the transcriptome of C42B‐ABi cells, thereby sustaining the Abiraterone resistance of C42B‐ABi cells both in vitro and in vivo. The BCL6 inhibitor significantly suppresses the expression of BCL6, NFIB and SMAD3 and inhibits the proliferation of C42B‐ABi cells. Additionally, BCL6 inhibitor BI‐3802 can synergize with Abiraterone to impede the growth of C42B‐ABi cells, suggesting that targeting this circuit is a potential strategy for treating Abiraterone‐resistant cases.

The accumulation of lipid droplets, that consist of a central core of triglycerides and cholesteryl esters, is caused by aberrant lipid metabolism. Lipids and cholesterol serve as precursors for steroid hormone synthesis, increasing intratumoral androgen production and promoting Enzalutamide resistance in CRPC cells.^[^ ^44^, ^45^ ^]^ These results suggest that the dysregulation of cholesterol homeostasis could be a common signature of resistance to androgen deprivation therapy. However, in our study, we found that the SE‐driven TF loop sustains drug resistance via increasing intratumoral cholesterol, testosterone, and dihydrotestosterone in Abiraterone‐resistant CRPC. Cholesterol, as an important component of cell membranes, can participate in regulating cell proliferation and other processes. At the same time, it serves as a precursor for the steroid hormones testosterone and dihydrotestosterone, regulating the level of androgen and thus activating the AR signaling. Meanwhile, we found that AR expression was lower in C42B‐ABi cells than C42B cells, a phenomenon resembling AR‐independent transdifferentiation or NEPC‐like dedifferentiation was observed.^[^ ^6^ ^]^ Knocking down BCL6, SMAD3 and NFIB could restore AR expression, suggesting that the C42B‐ABi cells regained sensitivity to Abiraterone, as our results confirmed (Figure 4H). These results suggest that TF mediates cell sensitivity to Abiraterone by regulating both intratumoral cholesterol homeostasis and AR expression.

Furthermore, our research identified this circuit enhances cholesterol synthesis of CRPC cells both in vitro and in vivo. Specifically, the circuit directly activates the transcription of key cholesterol synthesis genes including SREBF2, HMGCR, SCAP and FDFT1. SREBF2, as a transcription factor, activates the expression of HMGCR and SCAP, which are validated in C42B‐ABi cells. In C42B‐ABi cells, the overexpression of SREBF2 can counteract the proliferation inhibition caused by the knockdown of BCL6, NFIB, and SMAD3. Additionally, this circuit maintains resistance by regulating the cell cycle, with knockdown of these transcription factors resulting in cell cycle arrest at the G0/G1 phase. Mechanistically, our studies revealed that the circuit directly transcriptionally activates the G0/G1 cyclin‐dependent protein CDK2 and CCND3, thereby promoting cell cycle progression and the proliferation of C42B‐ABi cells (Figure 6E,F). Perturbation of cholesterol synthesis via SREBF2 knock‐down or HMGCR inhibition leads to cell cycle arrest in G0/G1 phase (Figure 6H,I). Therefore, we propose that the transcriptional regulatory circuit promotes the synthesis of cholesterol, testosterone, and dihydrotestosterone, by a SREBF2 mediated manner activating the transcription of cholesterol synthesis genes in some extent, to sustaining Abiraterone resistance. Secondly, This circuit affects the progression of cell cycle by regulating cholesterol synthesis, thereby promoting cell resistance. Furthermore, the statins and SREBF2 inhibitors can augment the cytotoxic effects of Abiraterone on C42B‐ABi cells, suggesting that targeting the downstream target genes of this circuit could be a potential therapeutic strategy for overcoming Abiraterone resistance. In particular, BCL6‐targeted inhibitors hold significant promise for clinical application. Multiple clinical‐stage inhibitors show promise, BMS‐986458 (Bristol‐Myers Squibb): Phase I trial (NCT06090539) as monotherapy and with rituximab, ARV‐393 (Arvinas): PROTAC degrader in Phase I (NCT06393738) for NHL.

## 4 Conclusion

In this study, we have unveiled the mechanisms by which aberrant activation of transcriptional regulation sustains Abiraterone resistance. Our research identifies and validates a transcriptional regulatory circuitry, driven by super‐enhancers composed of BCL6, NFIB and SMAD3, that promotes the development of Abiraterone resistance. Targeting this circuit or its downstream genes is proposed as a potential strategy for treating Abiraterone‐resistant CRPC.

## 5 Experimental Section

### Cell Lines and Patient Samples

C42B, PC3, 22RV1 and 293T cell lines were obtained from ATCC (American Type Culture Collection). Abiterone resistant cell lines, defined as C42B‐ABi, are estabolised by stepwise incubate the parental cell line C42B with increasing doses of Abiraterone, from 5 to 50 µmol L^−1^. To maintained the resistance, 10 µmol L^−1^ Abiterone routinely supplemented in the culture medium. C42B and C42B‐ABi were maintained in RPMI 1640 medium (Gbico). 293T was maintained in DMEM (Gbico). All media were supplemented with 10% fetal bovine serum (FBS, ExCell) and 1% penicillin‐streptomycin. All cells were cultured at 37 °C in an incubator containing 5% CO_2_.

The adjacent, PC and CRPC tissue in this study were obtained from the Department of Urology, the Third Affiliated Hospital of Guangzhou Medical University (Guangzhou, Guangdong, China), which was approved by the medical research ethics committee review (approve number: 2024‐008), with the permission of the patients.

### Antibodies and Reagents

The following antibodies purchased from CST were used in this study, includes anti‐BCL6 (#14 895), anti‐FLAG (#14 793), Rabbit (DA1E) mAb IgG XP Isotype Control (#3900), Mouse (G3A1) mAb IgG1 Isotype Control (#5415), anti‐CCND3 (#2936), anti‐CDK2 (#2546). Antibody of HMGCR (#13533‐1‐AP), NFIB (#29898‐1‐AP), SMAD3 (#30130‐1‐AP), SCAP (#12266‐1‐AP), SREBF2 (#28212‐1‐AP), FDFT1 (#13128‐1‐AP), AR (#5153), PSA (#5365) were purchased from Proteintech (Rosemont, USA). Antibody of H3K27ac (ab4729) was purchased from Abcam (Cambridge, MA). The molecules Abiraterone (S2246, Selleck), BI3802 (S6937, Selleck) were purchased from Selleck Chemicals and dissolved in DMSO and stored at −20 °C. The final dose of DMSO limited to 0.3% in all experiments.

### MTT Cell Proliferation Assay

The pre‐treated cell suspension was added to a 96‐well plate at 3000–4000 cells/100 µL medium (10% FBS)/well, and cultured for the indicated time course. Add 20 µL of MTT (3‐(4, 5‐dimethylthiazol–2‐yl)‐2, 5‐diphenyl tetrazolium bromide) to each well, incubate at 37 °C for 2 h, and measure the OD value at 490 nm on a microplate reader.^[^ ^46^ ^]^

### Cell Migration Assay

The edited cells were resuspended in 100 µL of serum‐free medium and seeded into a transwell chamber with an 8 µm diameter, at a density of 100 000 cells. The chamber was then placed in a 24‐well plate and supplemented with 600 µL of medium containing 10% FBS. After incubation for 24 h, the transwell chamber was removed, fixed and stained with crystal violet. Subsequently, images were captured under a microscope and cell counts were performed by Image J Software.

### Quantification of Total Cholesterol

1×10^^6^ cells were collected from each group, washed once with PBS, and then prepare cholesterol extract buffer chloroform: isopropanol: NP‐40 = 7:11:0.1. 200 µL cholesterol extract buffer was added to each sample, then vortex appropriately, centrifuged at 15 000 rpm for 10 min, discarded the precipitate, and the supernatant was retained and dried under vacuum. Finally, 200 µL assay buffer was added for dissolution. For serum total cholesterol measurement, 20 µL of serum was added to 180 µL Assay Buffer, and the mixture was mixed by blowing. For measurement of total cholesterol in tissues, 10 mg of tissue was crushed in a homogenizer in cholesterol extract buffer, then following the cell extraction steps as mentioned. Cholesterol quantification was subsequently performed the steps of the kit (TR13421, Thermo Scientific).

### ELISA Assay of Testosterone and Dihydrotestosterone

For sample preparation, 1×10^^6^ cells were collected, washed twice with PBS, and then suspended in 100 µL PBS. The cells were then subjected to repeated freeze‐thaw cycles at −80 °C, sonicated, and centrifuged to obtain the supernatant. For mouse serum samples, blood was allowed to clot naturally for 20 min, then centrifuged at 3000 rpm for 20 min to carefully collect the supernatant. For mouse tissue samples, 10 mg of tissue was homogenized with a certain amount of PBS using a homogenizer. The supernatant was then obtained by centrifugation. The prepared samples were then subjected to the instructions provided in the kit for testosterone (Mouse, E‐OSEL‐M0003; Human, E‐OSEL‐H0007; Elabscience, Wuhan, China) and dihydrotestosterone (E‐EL‐0031, Elabscience, Wuhan, China).

### Cell Cycle Assay

A certain number of cells were harvested, washed twice with PBS, and resuspended in pre‐chilled 70% ethanol for 2 h. Cell pellets were washed twice with PBS buffer and stained with propidium iodide. Cell cycle distribution was detected on a flow cytometer, and the resulting data were analyzed on a FlowJo X.

### ChIP‐Seq and CUT&Tag Analysis

ChIP uses the method described before.^[^ ^21^, ^46^ ^]^ However, tumor tissue needs to be extracted for some special processing before standardized ChIP. The tumor tissue was the size of soybean, and it was completely crushed by using a homogenizer under 2–3 mL 1% paraformaldehyde (#28908, Thermo) immersion, and then centrifugally filtered under a 25 µm filter. The obtained filtrate can continue to be carried out according to the ChIP step. CUT&Tag was performed according to the manufacturer's instructions (TD903, Vazyme).

Raw reads were initially aligned to the hg19 reference genome using Bwa (version 1.2.2) with default settings [19 451 168]. Subsequently, the high‐quality mapped reads were extracted and sorted by SAMtools (version 1.3.1) using the “‐q 10” parameter [19 505 943]. PCR duplicates were then eliminated using Picard MarkDuplicates tool (version 1.136) and blacklist regions were filtered out utilizing bedtools (version 2.27.1).

For histone ChIP‐seq data, MACS2 (Model‐Based Analysis of ChIP‐Seq, version 2.1.2) was applied to call peaks with parameters “‐q 0.01–extsize  =  146 ‐nomodel ‐B” [18 798 982]. Bigwig files were then generated via deepTools bamCompare function (version 3.1.3) using parameters “–operation subtract –normalizeUsing CPM –extend Reads 146 –binSize 20” [24 799 436]. For Cut‐tag data, peaks were identified through MACS2 with options “‐broad ‐q 0.01”. Bigwig files were generated using deepTools bamCoverage function with default settings.

### Super‐Enhancer Analysis

The C42B and C42B‐ABi specific H3K27ac peaks were extracted according to the non‐overlapping regions. Rank Order of Super Enhancers (ROSE),^[^ ^12^ ^]^ a common tool for SE identification, was utilized to identify SEs enriched within each specific peak set. In summary, ROSE merged enhancer elements within a 12 kb range and organized them by decreasing intensities. SEs were then defined as stitching elements with a tangent slope showing an inflection point value of ≥1.

### RNA‐Seq Analysis

Raw reads were mapped to GRCh37 reference genome utilizing HISAT2 (version 2.2.0) and quantified by htseq‐count program (version 0.11.3) at default setting.^[^ ^47^ ^]^ Then FPKM was employed to normalize the raw read counts. Differentially expressed genes were identified by the DESeq2 package (version 1.44.0) with p‐value < 0.05 and absolute log2 fold‐change (siRNA vs control) > 0.584.^[^ ^48^ ^]^

### Analysis of TF Co‐Binding in Relation to Gene Expression

First, intersection analysis was performed using BEDTools to identify overlaps between H3K27ac‐enriched regions and binding sites of the three transcription factors. This classified H3K27ac regions into three groups, regions bound by only one transcription factor (Solo group), regions bound by two transcription factors (Dual group), regions bound by all three transcription factors (Trio group). Subsequently, the annotatePeaks.pl module from HOMER was applied to perform genomic region annotation and gene association analysis for all H3K27ac regions. Regions annotated as “promoter‐TSS” were defined as promoters, while others were classified as enhancers. Finally, expression trends of genes associated with H3K27ac regions in each group were calculated using C42B‐ABi cell line RNA‐seq data. Differential expression patterns between pairwise groups were statistically evaluated using t‐tests.

### RT‐qPCR

Total RNA was extracted using the kit purchased from Accurate Biology (Human, China), and purified RNA was reverse transcribed in Maxima H Minus cDNA Synthesis Master Mix (Thermo Fisher). Quantitative real‐time qPCR was performed on an AB7300 detection system (Applied Biosystems, Foster City, CA, USA) using gene‐specific primers and Power SYBR Green PCR Master Mix (Applied Biosystems). Expression of each gene was normalized to GAPDH and quantified by the Comparative Ct method. RNA primer sequences are provided in Table S1 (Supporting Information).

### Immunoblotting Assay

Whole cell lysates were prepared in RIPA with added components.^[^ ^21^ ^]^ Immunoblots were performed using specific primary and HRP‐linked secondary antibodies as indicated.

### siRNA Knockdown

siRNA sequences were purchased from Shanghai Genechem Co or Gemma. Cells were transfected with 100 nm siRNA in OPTIMEM‐I (Gibico) for 24–48 h using Lipofectamine RNAiMAX transfection reagent (Invitrogen). siRNA sequences are provided in Table S2 (Supporting Information).

### Retroviral Infections

The BCL6, SMAD3, NFIB, SREBF2 expression vector was amplified based on the pCDH‐CMV‐Flag‐EF1‐puro vector, and 3xFLAG‐tag was added by PCR. The pLKO.1‐puro vector expressing shRNA targeting BCL6, NFIB and SMAD3 was constructed. To produce viral particles, the recombinant viral vector and packaging vector were co‐transfected into 293T cells. Viral particle‐containing supernatant was collected at 48 h and filtered through a 0.45 µm filter after transfection. Cells of interest were then infected with the virus under 10 µg mL^−1^ polybrene for 24–48 h. shRNA sequences are provided in Table S2 (Supporting Information).

### Dual Luciferase Reporter Assay

Experimental procedures were adapted from the previous studies.^[^ ^21^ ^]^ Candidate regions were amplified including the SMAD3 promoter (SMAD3‐P) and three enhancer regions (SMAD3‐CE1, SMAD3‐CE2, SMAD3‐CE3). The promoter region was cloned into the pGL3‐Basic firefly luciferase reporter vector, and each enhancer region was cloned into the pGL3‐Promoter firefly luciferase reporter vector (IGEbio). C42B‐ABi cells were transfected with BioT Transfection Reagent (Bioland, B01‐00). Co‐transfected with Renilla luciferase control vector as a normalization control. Appropriate control vectors (pGL3‐Promoter or pGL3‐Basic) were included in each experiment. Forty‐eight hours after transfection, lysed cells were collected using the Dual Luciferase Reporter Assay Kit (YEASEN, 11402ES) and luciferase activity was determined by a full wavelength reader (GloMax Discover).

### Lipid Droplet Staining Assay

Prepare a cell suspension at a density of 5×10^4^ to 1×10^5^ cells. Discard the culture medium and gently rinse the cells with PBS. Fix the cells with 4% paraformaldehyde (PFA) at room temperature for 10 min. Wash the cells twice with PBS to remove residual PFA. Prepare the staining solution by diluting Lipid dye (invitrogen, H34476) in PBS at a 1:1000 ratio (e.g., 1 µL dye in 1 mL PBS). Add 200–500 µL of the staining solution (enough to fully cover the cells) and mix thoroughly by gentle pipetting. Incubate at room temperature in the dark for 30 min. Fluorescence was quantified by flow cytometry (CytoFLEX) and visualized by microscopy (ZEISS).

### Xenograft Assays in Nude Mice

The animal experiments were subjected to the approval of the Institutional Animal Care and Use Committee of Guangdong Zhiyuan Biomedical Technology Co., LTD (Guangzhou, Guangdong, China) and following the ethical rules of animal experiments (approve number: AL20211205). 27 3‐week‐old nude mice were prepared, 24 of which underwent bilateral total orchiectomy (castrated group), and the remaining three groups were non‐castrated groups. Two weeks after orchiectomy, castrated mice were randomly divided into four groups, including PLKO, shBCL6, shSMAD3 and shNFIB, then subcutaneously inoculated with corresponding tumor cells. When the largest tumor diameter was ≈1.0 cm, Peripheral blood was collected from eyeballs for hormone detection, and tumors were dissected, weighted, and analyzed.

### Immunohistochemical staining

Paraffin sections of 4% paraformalin‐fixed tumor tissues were prepared according to the standard protocol for the detection of BCL6, SMAD3 and NFIB expression. Freshly configured DAB buffer (Beyotime, Shanghai, China) was used for the color reaction. Morphological HE staining was performed with hematoxylin. The prepared slides were scanned and photographed.

### Gene Set Enrichment Analysis (GSEA)

For the knockdown of BCL6, NFIB and SMAD3, the down‐regulated genes were individually chosen as the library genes. Then GSEAPreranked was performed using the fold change values from the knockdown of the respective TFs compared to the control as the ranked list. The significant terms with q value < 0.05 were shown.

### Survival Analysis

Taking BCL6, NFIB and SMAD3 as a signature, single‐sample GSEA was conducted to derive the enrichment score for each sample.^[^ ^49^ ^]^ To obtain the most significant relation between enrichment score and survival outcome, the optimal cutpoint was identified using the maximally selected rank statistics from the “maxstat”^[^ ^50^ ^]^ R package, followed by performing the log‐ranked test.

### Statistical Analysis

Two‐tailed Student's t‐test was used for the statistics of differences in measurement data between groups, Two‐way ANOVA Dunnett's multiple comparisons test was used for statistics of differences in measurement data between multiple groups, and One‐way ANOVA Dunnett's multiple comparisons test or t test was used for statistics of differences in count data between groups. All statistics and graphs were created with GraphPad Prism 8.0. Differences were considered statistically significant at P < 0.05 (*), P < 0.01 (**), P < 0.001 (***) and P<0.0001 (****); ns, not significant.

### Ethics Approval Statement

The adjacent, PC and CRPC tissue in this study were obtained from the Department of Urology, the Third Affiliated Hospital of Guangzhou Medical University (Guangzhou, Guangdong, China), which was approved by the medical research ethics committee review (approve number: 2024‐008), with the permission of the patients. The animal experiments were subjected to the approval of the Institutional Animal Care and Use Committee of Guangdong Zhiyuan Biomedical Technology Co., LTD (Guangzhou, Guangdong, China) and following the ethical rules of animal experiments (approve number: AL20211205).

## Conflict of Interest

The authors declare no conflict of interest.

## Author Contributions

L.L.J., J.M.W., G.J.P., and H.C.Z. contributed equally to this work as co‐first authors. X.P.S., Z.G.Z., and Y.Y.Z. conceived and directed the study as co‐corresponding authors. L.L.J., J.M.W., X.Y.L., and H.C.Z. performed the experiments. J.X.F., E.Z.L., and Y.Z.L. analyzed and interpreted the data. G.J.P., B.Y.L., Q.M., and L.Z.J. provided bioinformatics analysis. W.D., Y.Y.G., S.W.H., A.C.L., and Q.M.M. provided statistical and technical support. Z.G.Z. and J.M.W. were responsible for providing clinical advice. L.L.J. and H.C.Z. wrote the first draft of the manuscript. X.P.S. and Y.Y.Z. revised the manuscript. All the authors had full access to the data, and reviewed the manuscript before submission.

## Supporting information



Supporting Information

Supporting Information

Supporting Information

## Data Availability

The data that support the findings of this study are available on request from the corresponding author. The data are not publicly available due to privacy or ethical restrictions.
