# Supplementary material for: A Super‐Enhancer‐Driven Transcriptional Regulatory Circuit Underlying Abiraterone Resistance in Castration‐Resistant Prostate Cancer
Source: Adv Sci (Weinh). 2025 Jun 5;12(31):e01284. doi: 10.1002/advs.202501284 (PMC12376675; doi:10.1002/advs.202501284)
Supplement: Supplementary file 1 — Supporting Information [file ADVS-12-e01284-s002.docx]

**
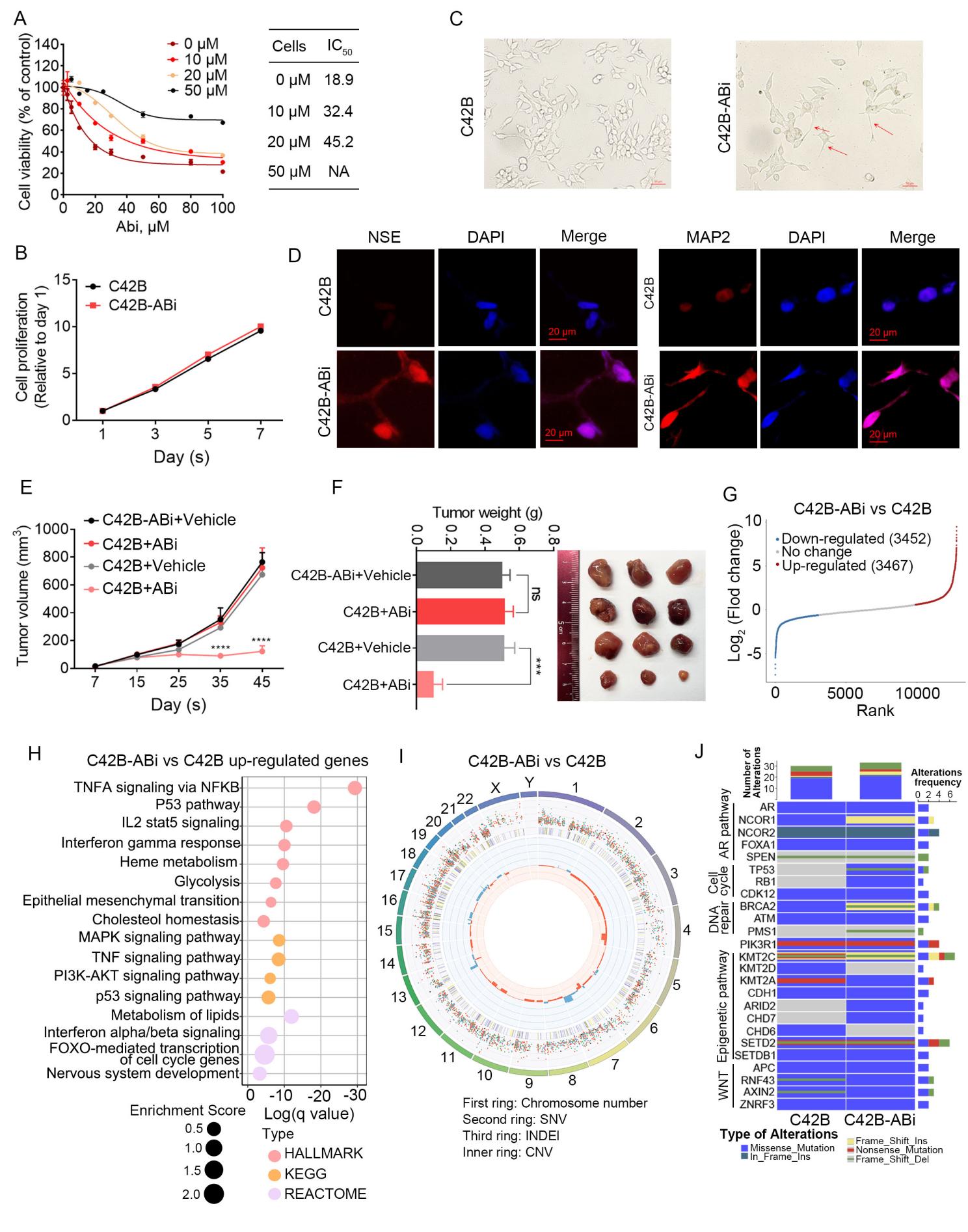
**

**Supplementary Figure 1**

**A,** Abiraterone dose-response curves for indicated C42B-ABi cell lines. Parental C42B cells (baseline sensitivity, 0 μM), intermediate-resistant clones (10/20 μM), and fully resistant C42B-ABi cells (50 μM). **B,** The relative cell proliferation of C42B and C42B-ABi cells were detected. **C,** The morphological comparison of C42B and C42B-ABi cells. **D,** Immunofluorescence analysis conducted to detect the expression differences of NSE and MAP2 in C42B and C42B-ABi cells . **E,** C42B-ABi cells or C42B cells were inoculated into castrated nude mice, with subsequent administration of ABi treatment or vehicle control, and tumor volume was monitored throughout the experimental period. Two-way ANOVA Dunnett’s multiple comparisons test. n = 6. ****P < 0.0001. **F,** Tumor weights were measured (left), and representative tumors were photographed (right) after mice were sacrificed. One-way ANOVA Dunnett’s multiple comparisons test. n = 6. ***P < 0.001. **G,** Visualization of differential gene expression between C42B and C42B-ABi cells. The X axis represents individual gene, while the Y axis portrays the log_2_ fold change in mRNA expression in C42B-ABi cells relative to C42B cells. Red/Blue dots highlight 3452/3467 genes upregulated/downregulated in C42B-ABi cells. **H,** GSEA, KEGG and REACTOME enrichment analysis based on upregulated genes in C42B-ABi cells . **I,** Whole exome sequencing comparison between C42B-ABi and C42B cells. **J,** Visualize the drug resistance-associated mutations in C42B-ABi cells.


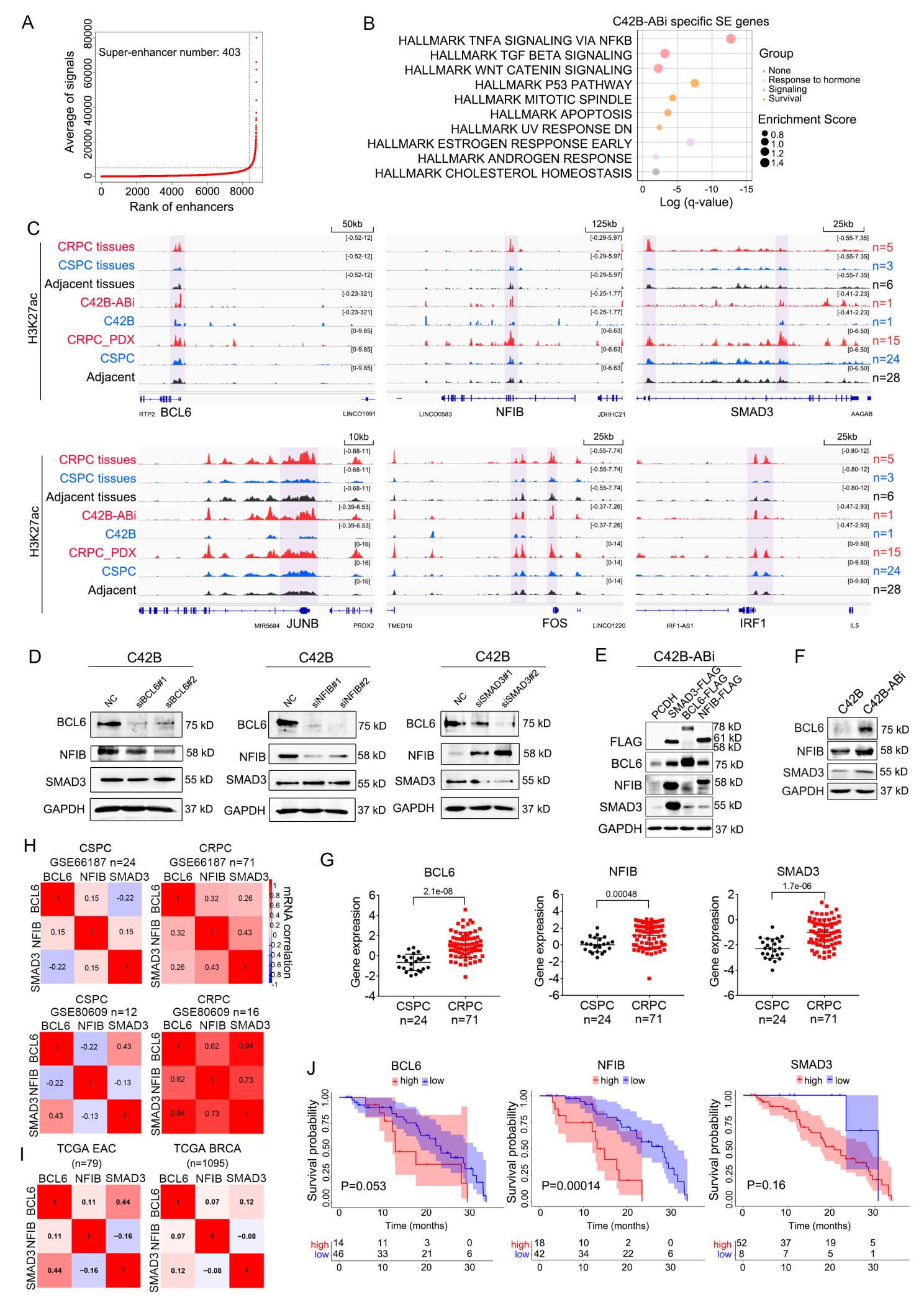


**Supplementary Figure 2**

**A,** C42B specific enhancer ranking plot based on H3K27ac ChIP-Seq signals using the ROSE algorithm. **B,** Pathway enrichment analysis for genes driven by C42B-ABi specific super-enhancers. **C,** IGV showing H3K27ac binding at the BCL6, NFIB, SAMD3, JUNB, FOS, and IRF1 loci upon indicated groups. **D,** Using siRNA to knock down BCL6, SMAD3, or NFIB in C42B, the protein expression of these three molecules was analyzed by Western blot. **E,** Overexpress flag-tagged BCL6, NFIB, and SMAD3 individually and the protein level of these molecules were examined. **F,** The basic expression of BCL6, NFIB, and SMAD3 in C42B and C42B-ABi cells. **G,** The mRNA expression of BCL6, NFIB and SMAD3 in patient samples of CSPC and CRPC (GSE66187). The t-test was used to determine statistical differences between two groups. **H,** Correlation analysis among the BCL6, NFIB, and SMAD3 in patient samples of CSPC and CRPC (GSE66187,GSE80609). **I,** Correlation analysis among the BCL6, NFIB, and SMAD3 across indicated tumor tissues from TCGA database. **J,** Kaplan-Meier analysis of cohort divided by the median expression value of BCL6, NFIB, and SMAD3 gene signature individually.


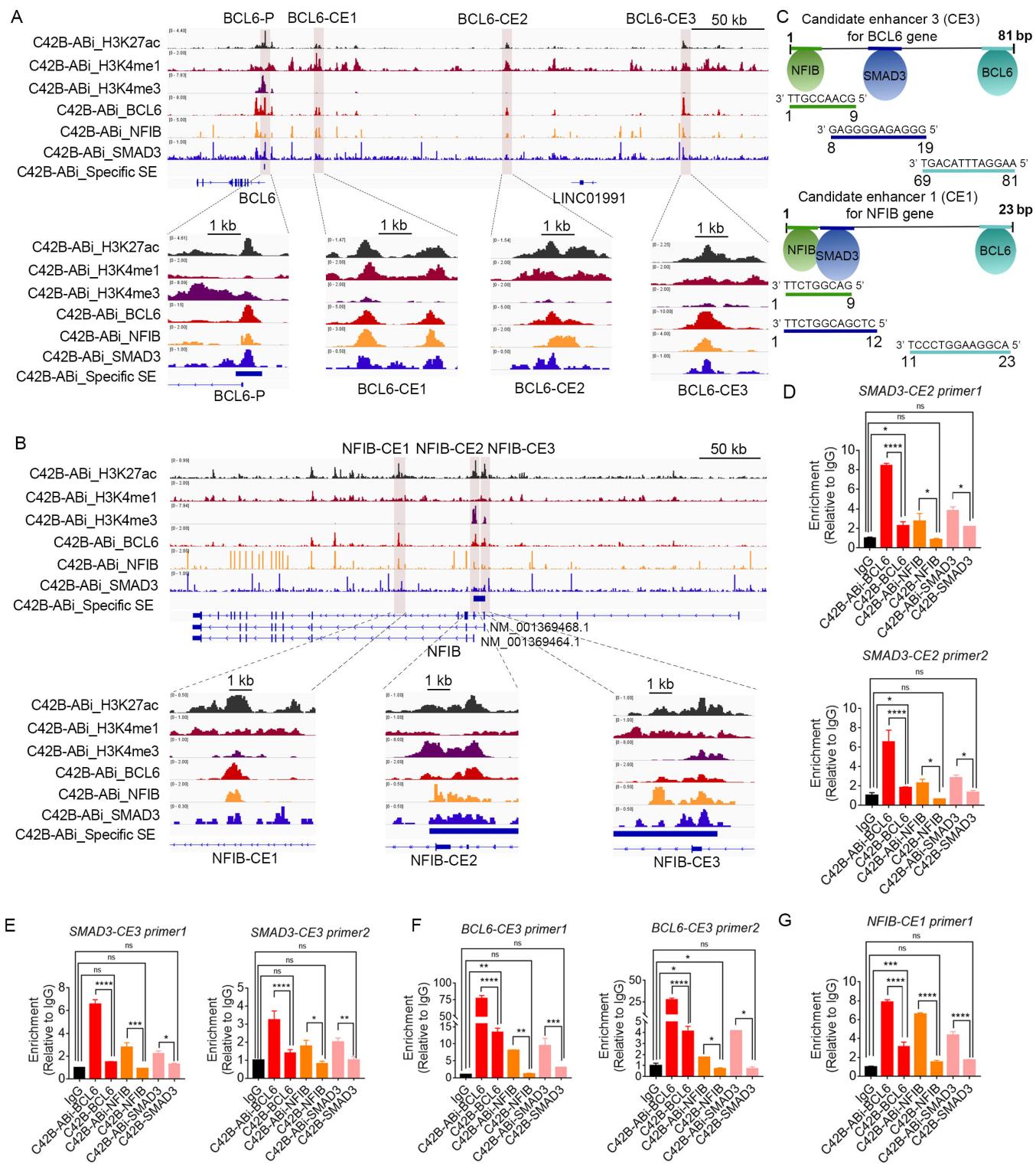


**Supplementary Figure 3**

**A, B,** IGV plots of CUT&Tag showing co-occupancy of BCL6, SMAD3, and NFIB at the promoter and enhancer of the BCL6 and NFIB gene locus. **C,** A schematic representation of the nearest distance pattern of BCL6, SMAD3 and NFIB motifs at the candidate enhancer location. **D, E, F, G,** ChIP-qPCR targeting SMAD3-CE2 (D), SMAD3-CE3 (E), BCL6-CE3 (F) , NFIB-CE1 (G) was performed on C42B and C2B-ABi cells using indicated antibody. IgG was used as a negative control antibody. One-way ANOVA Dunnett’s multiple comparisons test. n=3. *, P <0.05; **, P < 0.01; ***, P < 0.001; ****, P < 0.0001.


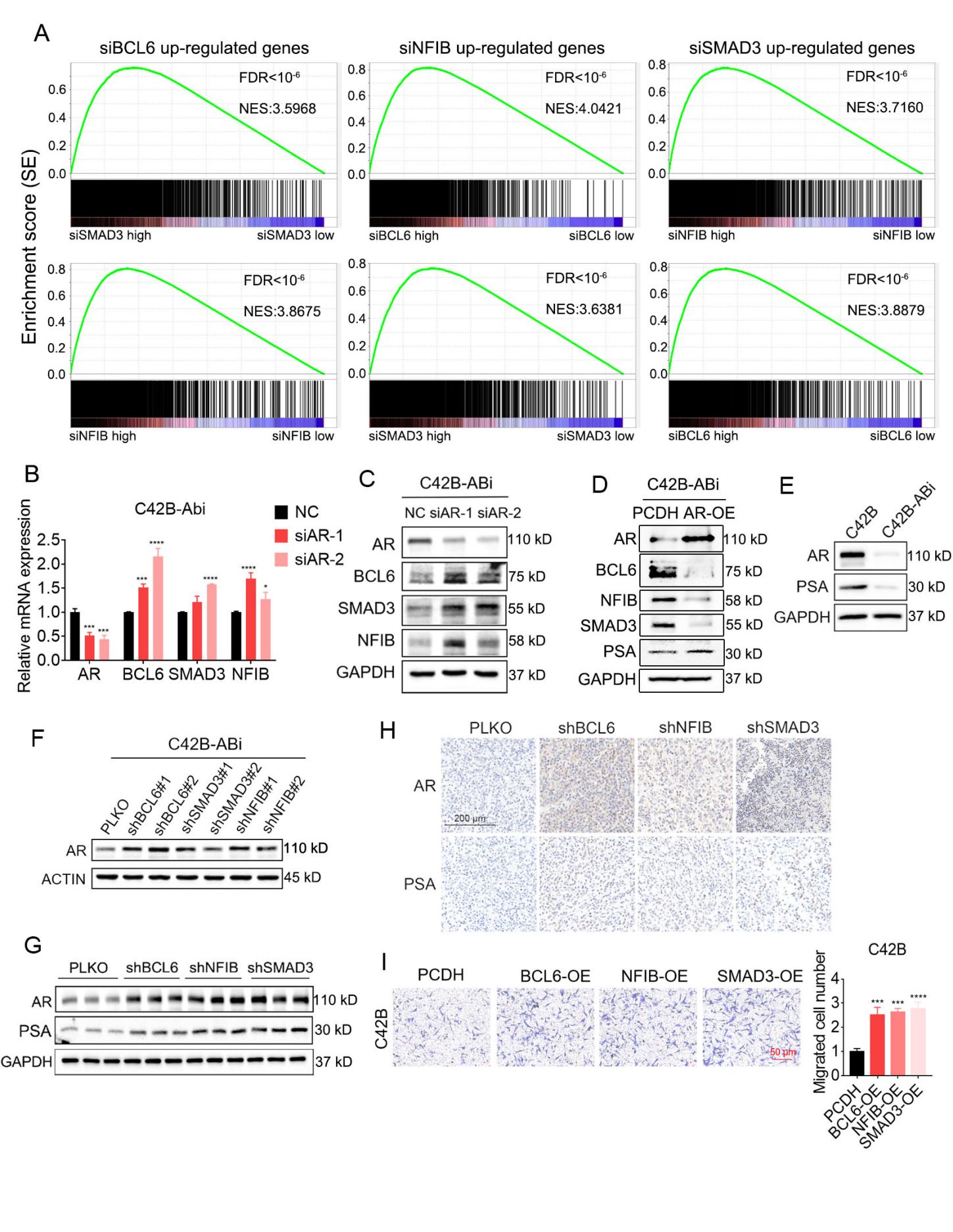


**Supplementary Figure 4**

**A,** Genes up-regulated by knocking down BCL6 are subjected to GSEA enrichment analysis with the RNA-Seq data of SMAD3 or NFIB knockdown. Similarly, up-regulated genes from the SMAD3 or NFIB knockdown are analyzed in the same manner. **B, C,** Silencing AR by siRNA in C42B-ABi cells, the mRNA (B) and protein (C) level expressions of AR, BCL6, NFIB, and SMAD3 were detected. Two-way ANOVA Dunnett’s multiple comparisons test. n = 3. *, P <0.05; ***, P < 0.001; ****, P < 0.0001. **D,** The protein expression of AR, PSA, BCL6, NFIB, and SMAD3 in the control and C42B-Abi with the over-expression of AR. **E,** The protein level of AR and PSA in C42B and C42B-ABi cells. **F,** The protein level of AR in C42B-ABi cells with BCL6, SMAD3, and NFIB knockdown. **G,** The protein level of AR and PSA in tumour tissue were detected by Western blots. **H,** Immunohistochemical (IHC) analysis of the indicated proteins in tumour tissue. **I,** The cell migration of C42B with the over-expression of BCL6, NFIB and SMAD3 was detected. One-way ANOVA Dunnett’s multiple comparisons test. n = 3. ***, P < 0.001; ****, P < 0.0001.


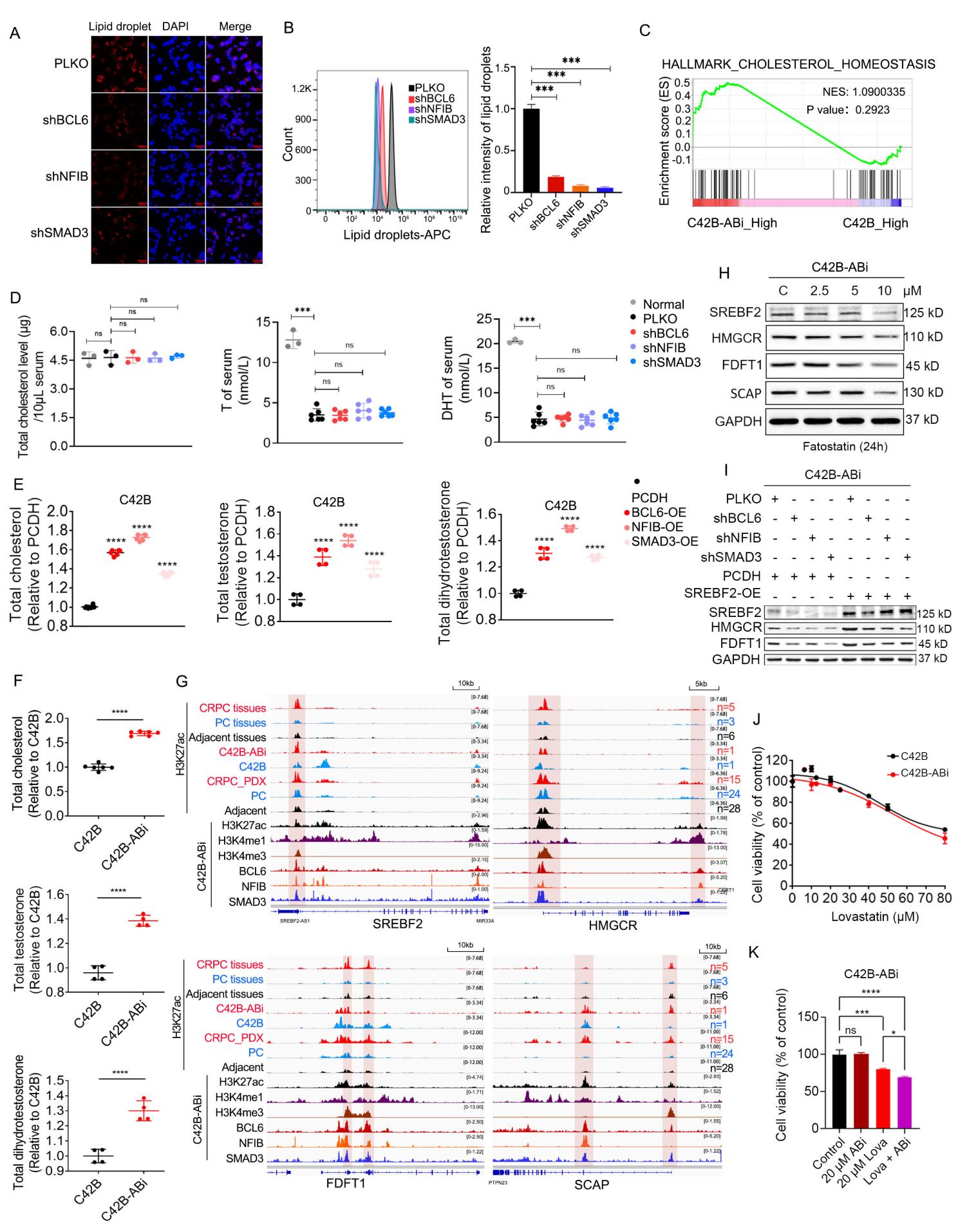


**Supplementary Figure 5**

**A,** Immunofluorescence analysis conducted to detect lipid droplet staining in C42B-ABi cells with BCL6, SMAD3, or NFIB silenced. **B,** Silencing BCL6, NFIB, and SMAD3 by shRNA in C42B-ABi cells, the intensity of lipid droplets were detected by flow cytometry. One-way ANOVA Dunnett’s multiple comparisons test. n = 3. ***, P < 0.001. **C,** The gene sets associated with cholesterol homeostasis was subjected to GSEA enrichment analysis with the RNA-Seq data of C42B-ABi and C42B. **D,** ELISA assay measured the levels of cholesterol, testosterone (T), and dihydrotestosterone (DHT) in mice serum. One-way ANOVA Dunnett’s multiple comparisons test. n = 3. ***, P < 0.001; ****, P < 0.0001. **E,** Intracellular cholesterol, testosterone (T), and dihydrotestosterone (DHT) levels measured in C42B cells with BCL6, SMAD3 or NFIB over expression. One-way ANOVA Dunnett’s multiple comparisons test. n = 3. ***, P < 0.001; ****, P < 0.0001. **F,** Intracellular cholesterol, testosterone (T), and dihydrotestosterone (DHT) levels measured in C42B and C42B-ABi cells. One-way ANOVA Dunnett’s multiple comparisons test. n = 3. ***, P < 0.001; ****, P < 0.0001. **G,** IGV showing H3K27ac, H3K4me1, H3K4me3, BCL6, NFIB, and SMAD3 binding at the SREBF2, HMGCR, FDFT1 and SCAP loci upon indicated groups. **H,** The protein expressions of SREBF2, HMGCR, FDFT1 and SCAP were detected with fatostatin (SREBF2 inhibitor) treatment. **I**, Overexpress SREBF2 after silencing BCL6, SMAD3, and NFIB by shRNA in C42B-ABi cells, the protein expressions of SREBF2, HMGCR, FDFT1 and SCAP was detected. **J,** Lovastatin (HMGCR inhibitor) dose-response curves for C42B and C42B-ABi cells. **K,** C42B-ABi cells incubated with Abiraterone or/and lovastatin, the cell proliferation was detected by MTT assay. One-way ANOVA Dunnett’s multiple comparisons test. n = 3. *, P < 0.05; ***, P < 0.001; ****, P < 0.0001.

**Supplementary table 1. qPCR primers of target genes**

| Genes | Forward (5'- 3') | Reverse (5'- 3') |
| --- | --- | --- |
| SMAD3-Homo | TGACCACCAGATGAACCAC | ACATTGGAGAGCAGCCCTA |
| NFIB-Homo | CCATCTGTCTCACTCAGGATGAA | TGCACGGACATGTGGAAGAA |
| JUNB-Homo | CCACCTCCCGTTTACACCAA | GAGGTAGCTGATGGTGGTCG |
| SCAP-Homo | TATCTCGGGCCTTCTACAACCA | ACACAACTCCTCCAAGCTCCTG |
| GAPDH-Homo | GGACCTGACCTGCCGTCTAG | GTAGCCCAGGATGCCCTTGA |
| BCL6-Homo | CAGTCCCCACTCACTCACAT | AGGCCATTTTGTCTTCACCAAT |
| SREBF2-Homo | TTGTCGGGTGTCATGGGC | ACAAATTGCAGCATCTCGTCG |
| FDFT1-Homo | ACTCGACAGACTCTAAGGCTC | CTTCCTCCCCACTTGGTCAAT |
| LDLR-Homo | AGCTACCCCTCGAGACAGAT | ACTGTCCGAAGCCTGTTCTG |
| HMGCR-Homo | AGTGAGATCTGGAGGATCCAA | CCCCACTATGACTTCCCAGG |
| FOS-Homo | GGGGCAAGGTGGAACAGTTAT | CAGGTTGGCAATCTCGGTCT |
| ELF3-Homo | GATCTCCGAGCAAGAGCGTAG | CGAGCTGTACATCGCACTGA |
| AR-Homo | CCTGGCACACTCTCTTCACA | GGATAGGGCACTCTGCTCAC |
| KLK3-Homo | GTGATGACTCCAGCCACGACCTC | CATGGAGGTCCACACACTGAAG |
| CCL2-Homo | CAGCCAGATGCAATCAATGCC | TGGAATCCTGAACCCACTTCT |
| E2F1-Homo | AGCGGCGCATCTATGACATC | GTCAACCCCTCAAGCCGTC |
| TK1-Homo | GGGCAGATCCAGGTGATTCTC | TGTAGCGAGTGTCTTTGGCATA |
| CDKN2D-Homo | GCCTTCTGCACCGGGAG | TGCCAAACATCATGACCTGC |
| CDK2-Homo | CCAGGAGTTACTTCTATGCCTGA | TTCATCCAGGGGAGGTACAAC |
| CCND3-Homo | GACCGACAGGCCTTGGTCAA | AGTGCCAGTGATCCCTGCCA |

**Supplementary table 2**

**siRNA sequence of target genes**

| Genes |  | Sense (5'-3') | Antisense (5'-3') |
| --- | --- | --- | --- |
| AR-Homo | si#1 | GACAGUGUCACACAUUGAAdTdT | UUCAAUGUGUGACACUGUCdTdT |
|  | si#2 | GGAAAGUCAAGCCCAUCUAdTdT | UAGAUGGGCUUGACUUUCCdTdT |
| BCL6-Homo | si#1 | GCCACUCACCACUCUACAUdTdT | UGUAGUUCAUUCGAAGUUCdTdT |
|  | si#2 | CCCUAUCCCUGUGAAAUCUdTdT | AUGUAGAGUGGUGAGUGGCdTdT |
| FOS-Homo | si#1 | CCGAGCCCUUUGAUGACUUdTdT | AGAUUUCACAGGGAUAGGGdTdT |
|  | si#2 | GCAAGAUCCCUGAUGACCUdTdT | AAGUCAUCAAAGGGCUCGGdTdT |
| SMAD3-Homo | si#1 | GGAUGCAACCUGAAGAUCUdTdT | AGGUCAUCAGGGAUCUUGCdTdT |
|  | si#2 | CCGCAUGAGCUUCGUCAAAdTdT | AGAUCUUCAGGUUGCAUCCdTdT |
| NFIB-Homo | si#1 | CUGGAGUCAACUUCCCAAUdTdT | UUUGACGAAGCUCAUGCGGdTdT |
|  | si#2 | CCUUCCUACAUCAGCAACAdTdT | AUUGGGAAGUUGACUCCAGdTdT |
| SREBF2-Homo | si#1 | GCAAUUUGUCAGUAAUCAAdTdT | UGUUGCUGAUGUAGGAAGGdTdT |
|  | si#2 | GGCAGUGGUGGUAGUGGUAdTdT | UUGAUUACUGACAAAUUGCdTdT |
| JUNB-Homo | si#1 | CGACGACCACCAUCAGCUAdTdT | UACCACUACCACCACUGCCdTdT |
|  | si#2 | GCAUCAAAGUGGAGCGCAAdTdT | UAGCUGAUGGUGGUCGUCGdTdT |
| IRF1-Homo | si#1 | GCCGAGACACUAAGAGCAAdTdT | UUGCUCUUAGUGUCUCGGCdTdT |
|  | si#2 | UACCAGAUAGCACCACUGAdTdT | UCAGUGGUGCUA UCUGGUAdTdT |

**shRNA sequence of target genes**

| Genes |  | Sequence (5'-3') |
| --- | --- | --- |
| BCL6-Homo | sh#1 | CCGGCCGGCTCAATAACATCGTTAACTCGAGTTAACGATGTTATTGAGCCGGTTTTTGAATT |
|  | sh#2 | CCGGGCCTGTTCTATAGCATCTTTACTCGAGTAAAGATGCTATAGAACAGGCTTTTTGAATT |
| NFIB-Homo | sh#1 | CCGGGCACCCGTGCTGTGTCTTATCCTCGAGGATAAGACACAGCACGGGTGCTTTTTGAATT |
|  | sh#2 | CCGGGCACGAAAGAGATCAAGATATCTCGAGATATCTTGATCTCTTTCGTGCTTTTTGAATT |
| SMAD3-Homo | sh#1 | CCGGGGCTGCTCTCCAATGTCAACACTCGAGTGTTGACATTGGAGAGCAGCCTTTTTGAATT |
|  | sh#2 | CCGGGCAACCTGAAGATCTTCAACACTCGAGTGTTGAAGATCTTCAGGTTGCTTTTTGAATT |
