# Supplementary material for: A Super‐Enhancer‐Driven Transcriptional Regulatory Circuit Underlying Abiraterone Resistance in Castration‐Resistant Prostate Cancer
Source: Adv Sci (Weinh). 2025 Jun 5;12(31):e01284. doi: 10.1002/advs.202501284 (PMC12376675; doi:10.1002/advs.202501284)

ADVANCED SCIENCE
Open Access

Supporting Information

for *Adv. Sci.*, DOI 10.1002/advs.202501284

A Super-Enhancer-Driven Transcriptional Regulatory Circuit Underlying Abiraterone Resistance in Castration-Resistant Prostate Cancer

Liling Jiang, Jiamin Wang, Guanjie Peng, Haichuan Zhang, Jinxin Fang, Yingyin Gao, Enzhe Lou, Yangzhou Liu, Wa Ding, Bingyuan Liu, Qiong Mao, Lizhen Jiang, Aochu Liu, Xinyue Li, Shiwen Hu, Qiaomin Ma, Yueyuan Zheng, Zhigang Zhao* and Xianping Shi**

Figure 1H

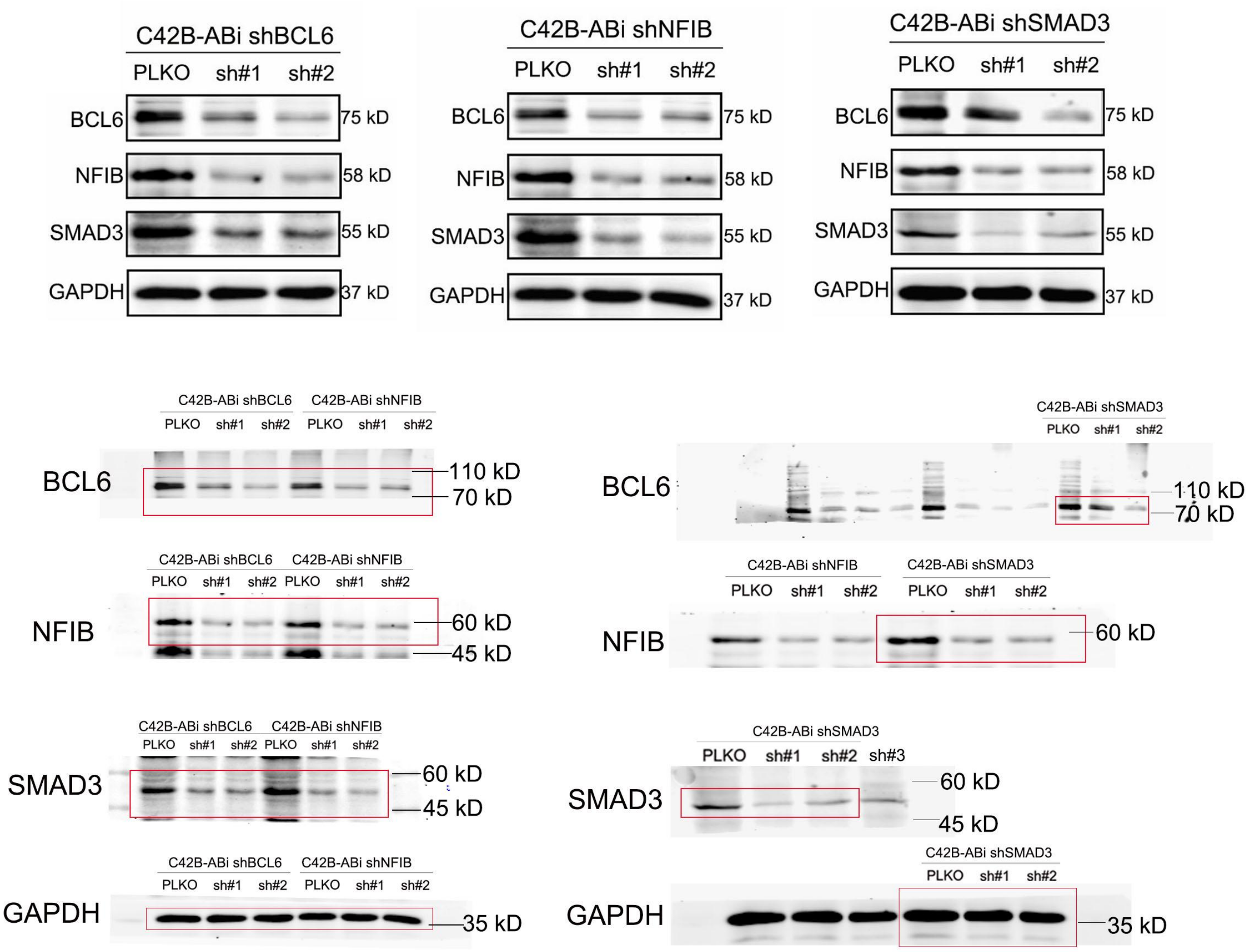

C42B-ABi shBCL6
PLKO sh#1 sh#2
BCL6 75 kD
NFIB 58 kD
SMAD3 55 kD
GAPDH 37 kD
C42B-ABi shNFIB
PLKO sh#1 sh#2
BCL6 75 kD
NFIB 58 kD
SMAD3 55 kD
GAPDH 37 kD
C42B-ABi shSMAD3
PLKO sh#1 sh#2
BCL6 75 kD
NFIB 58 kD
SMAD3 55 kD
GAPDH 37 kD
C42B-ABi shBCL6 C42B-ABi shNFIB
PLKO sh#1 sh#2 PLKO sh#1 sh#2
BCL6
110 kD
70 kD
C42B-ABi shSMAD3
PLKO sh#1 sh#2
BCL6
110 kD
70 kD
C42B-ABi shBCL6 C42B-ABi shNFIB
PLKO sh#1 sh#2 PLKO sh#1 sh#2
NFIB
60 kD
45 kD
C42B-ABi shNFIB C42B-ABi shSMAD3
PLKO sh#1 sh#2 PLKO sh#1 sh#2
NFIB
60 kD
C42B-ABi shBCL6 C42B-ABi shNFIB
PLKO sh#1 sh#2 PLKO sh#1 sh#2
SMAD3
60 kD
45 kD
C42B-ABi shSMAD3
PLKO sh#1 sh#2 sh#3
SMAD3
60 kD
45 kD
C42B-ABi shBCL6 C42B-ABi shNFIB
PLKO sh#1 sh#2 PLKO sh#1 sh#2
GAPDH
35 kD
C42B-ABi shSMAD3
PLKO sh#1 sh#2
GAPDH
35 kD

Figure 2F

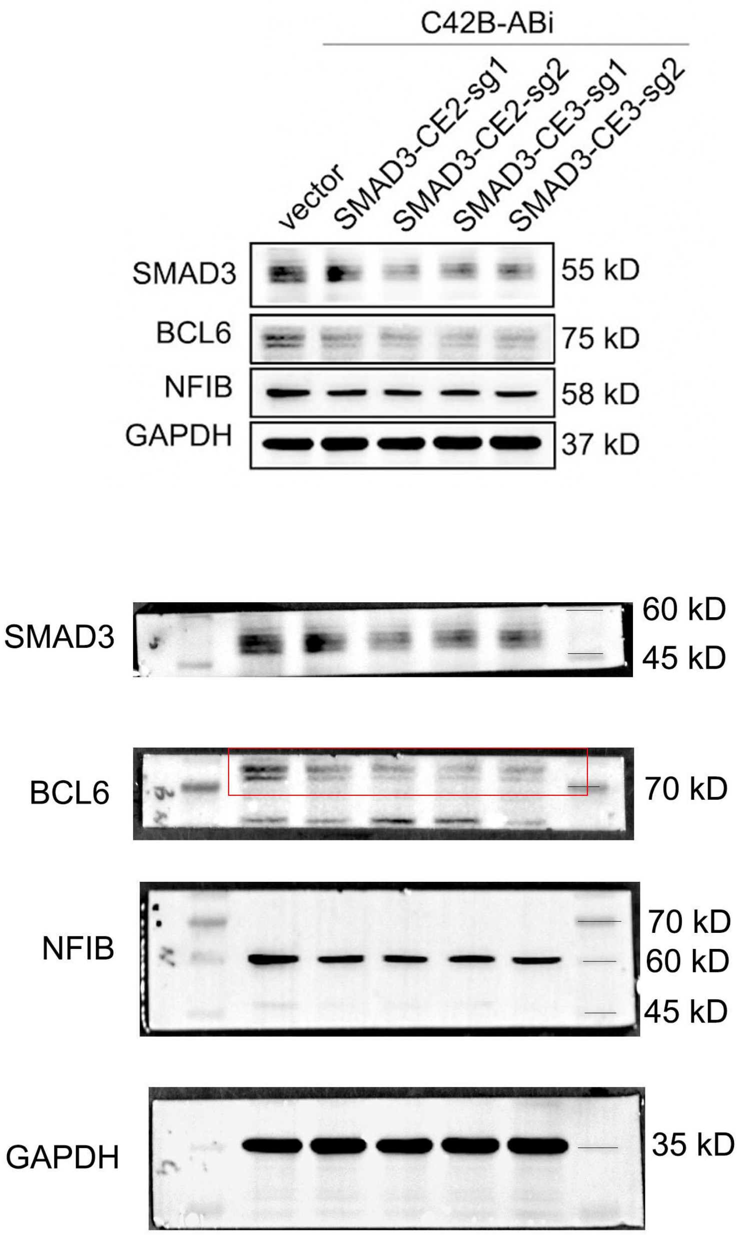

C42B-ABi
vector
SMAD3-CE2-sg1
SMAD3-CE2-sg2
SMAD3-CE3-sg1
SMAD3-CE3-sg2
SMAD3
55 kD
BCL6
75 kD
NFIB
58 kD
GAPDH
37 kD
SMAD3
60 kD
45 kD
BCL6
70 kD
NFIB
70 kD
60 kD
45 kD
GAPDH
35 kD

Figure 2J

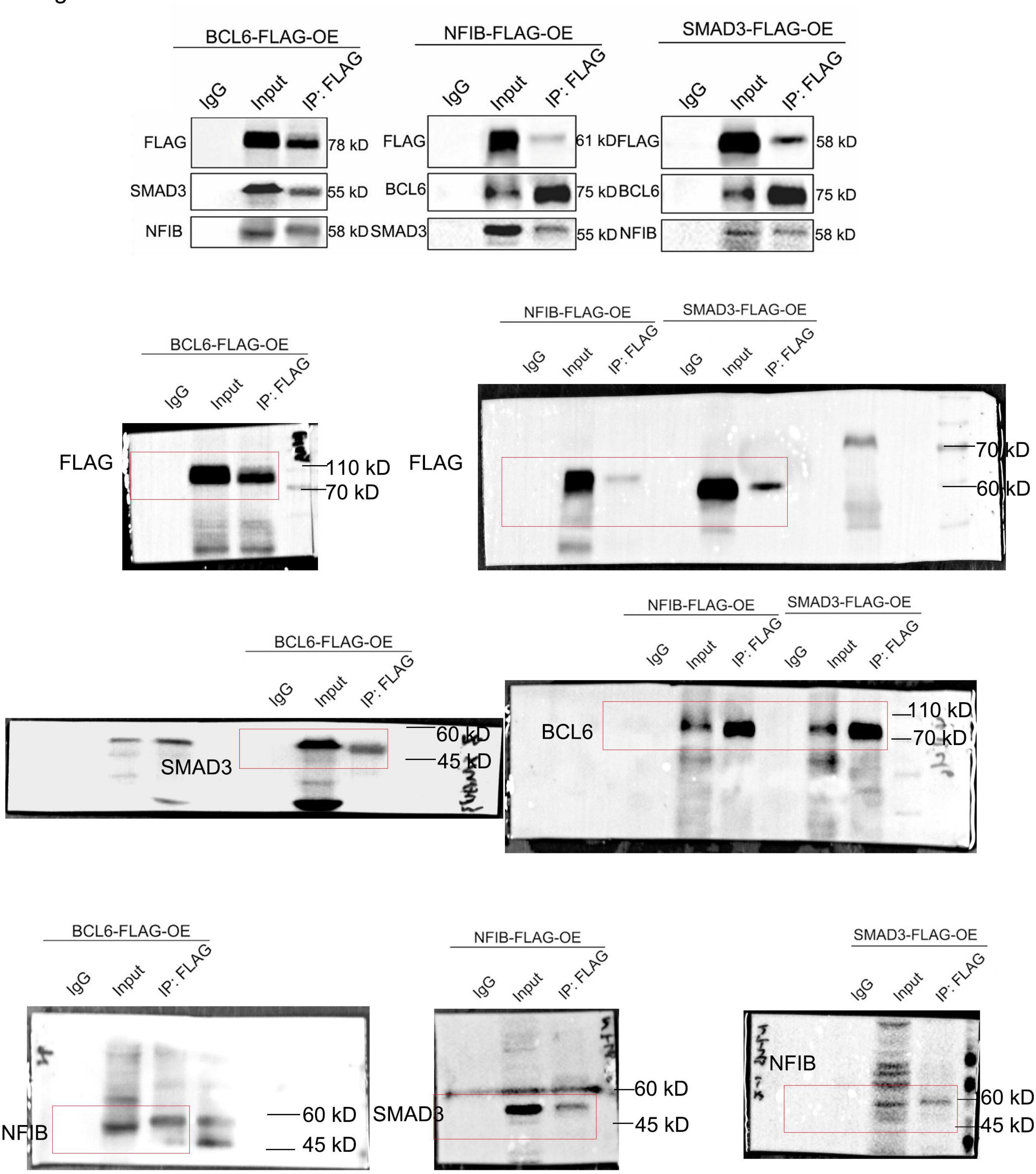

BCL6-FLAG-OE
NFIB-FLAG-OE
SMAD3-FLAG-OE
IgG
Input
IP: FLAG
FLAG
SMAD3
NFIB
BCL6
78 kD
55 kD
58 kD
61 kD
75 kD
110 kD
70 kD
60 kD
45 kD

Figure 4F

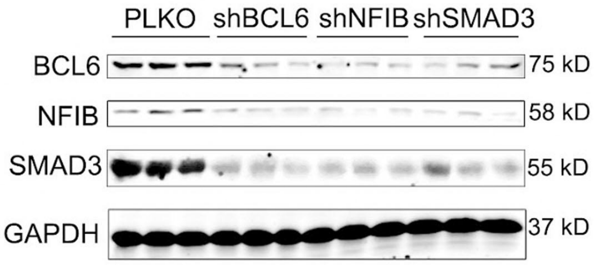
PLKO
shBCL6
shNFIB
shSMAD3
BCL6
NFIB
SMAD3
GAPDH
75 kD
58 kD
55 kD
37 kD

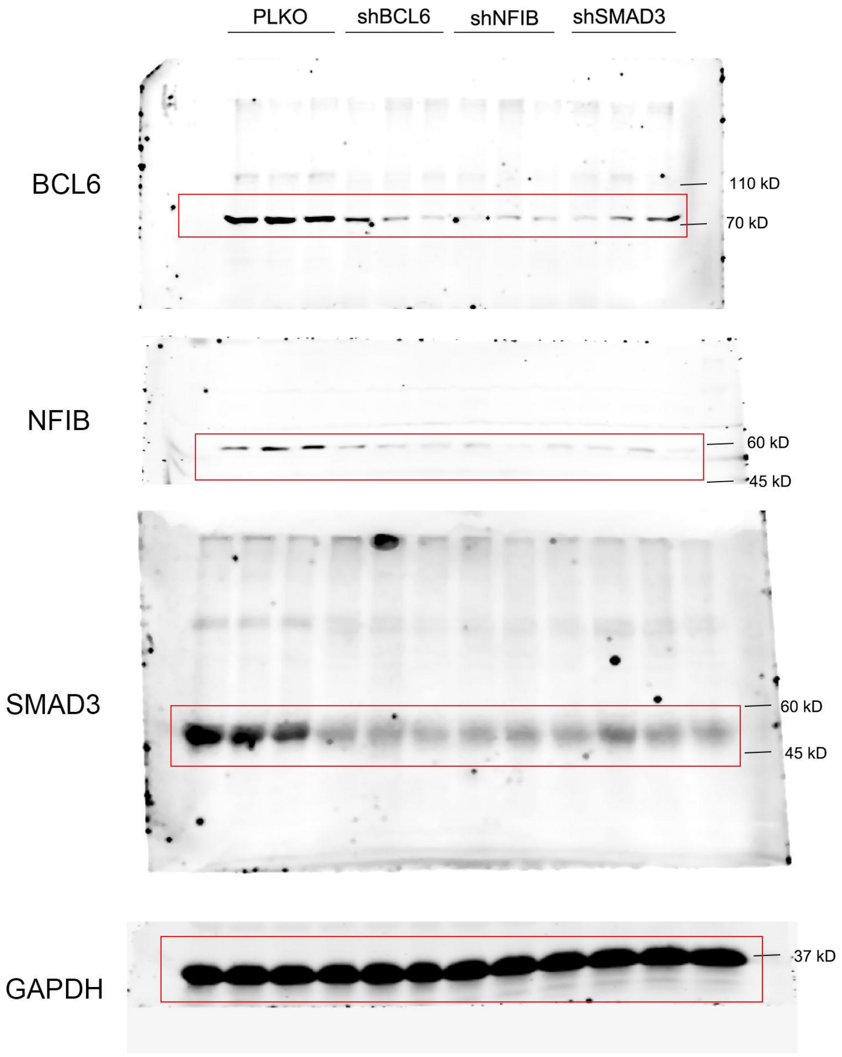
PLKO
shBCL6
shNFIB
shSMAD3
BCL6
110 kD
70 kD
NFIB
60 kD
45 kD
SMAD3
60 kD
45 kD
GAPDH
37 kD

Figure 4I

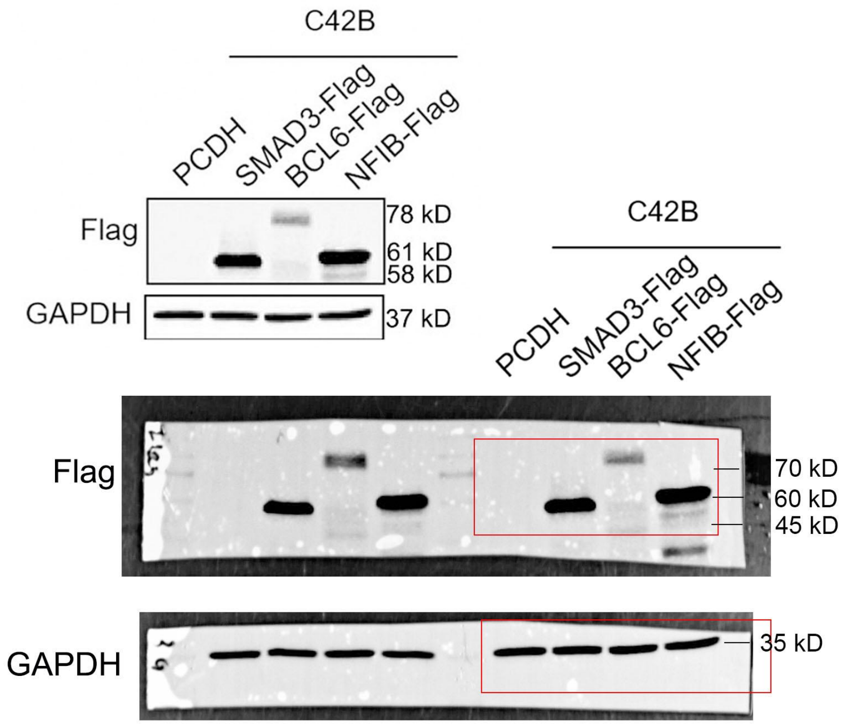

Figure 4M

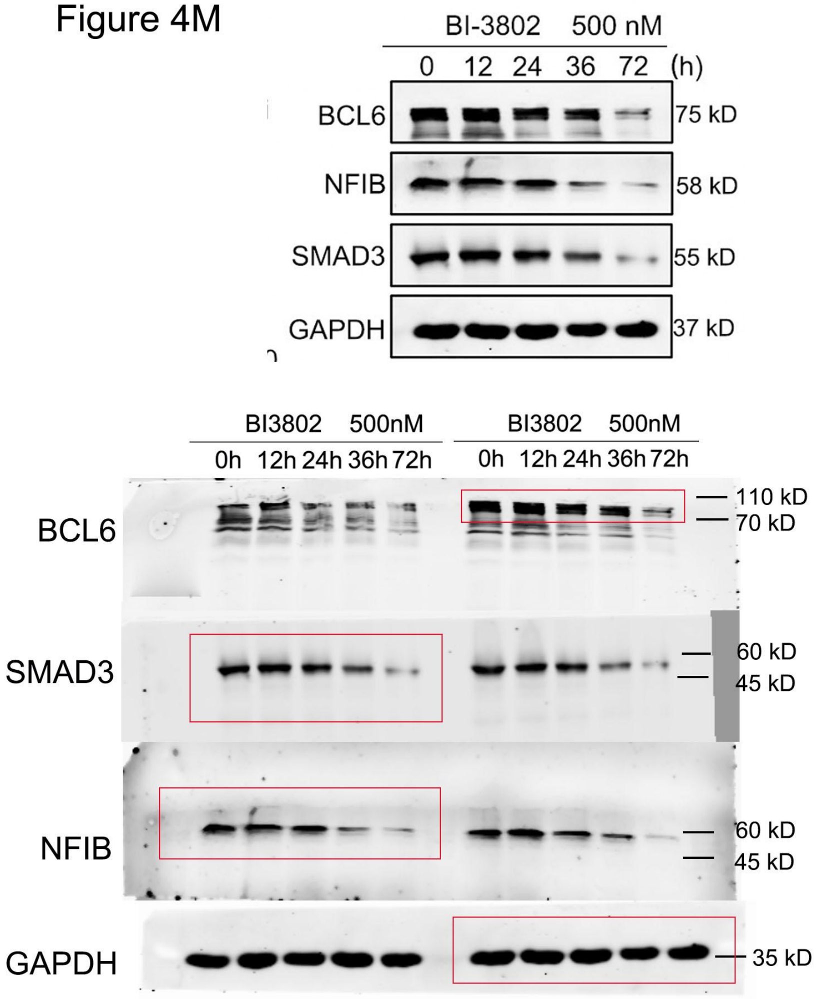

Figure 5G

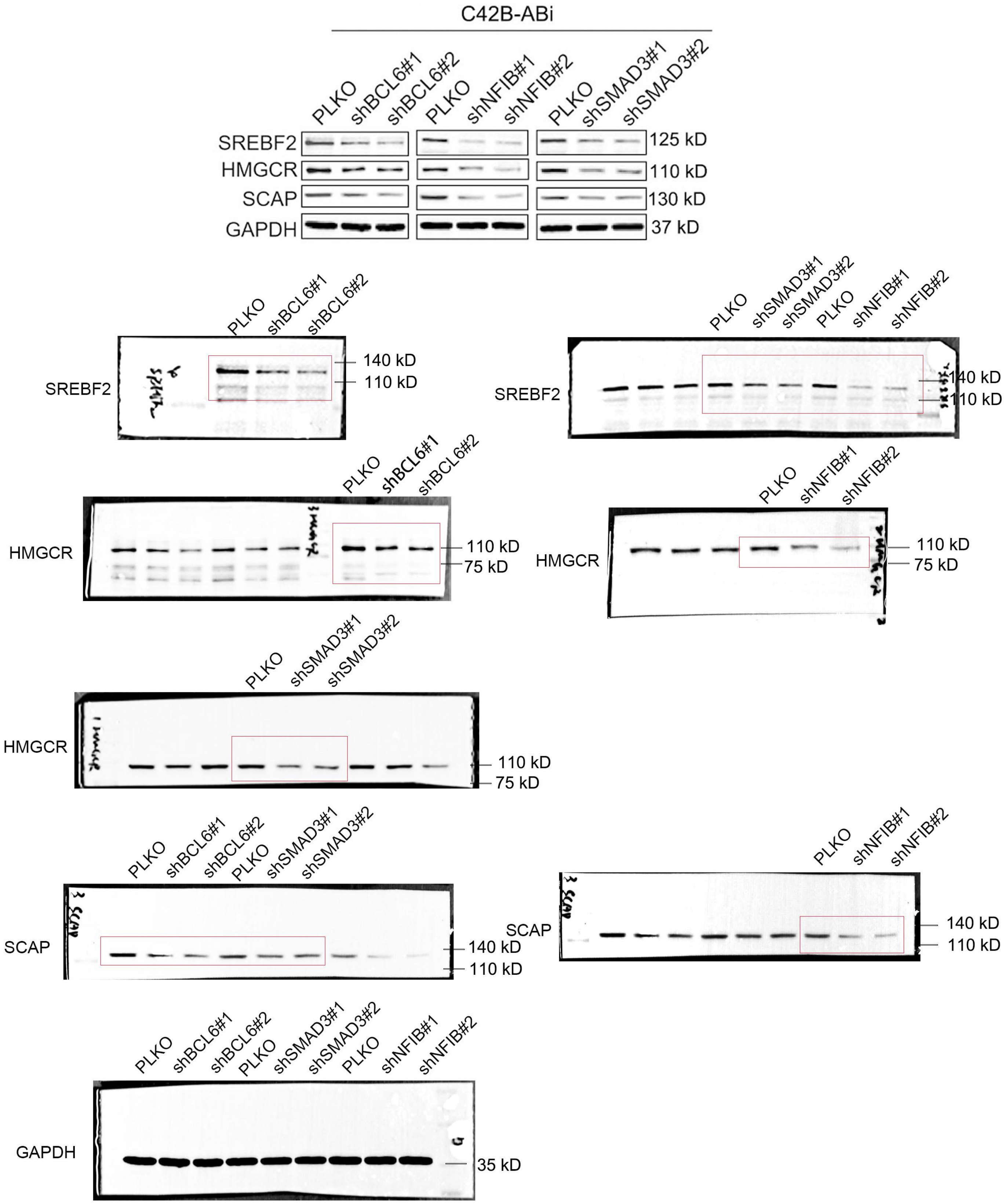
C42B-ABi
PLKO
shBCL6#1
shBCL6#2
PLKO
shNFIB#1
shNFIB#2
PLKO
shSMAD3#1
shSMAD3#2
SREBF2
HMGCR
SCAP
GAPDH
125 kD
110 kD
130 kD
37 kD
140 kD
110 kD
75 kD
35 kD

Figure 5H

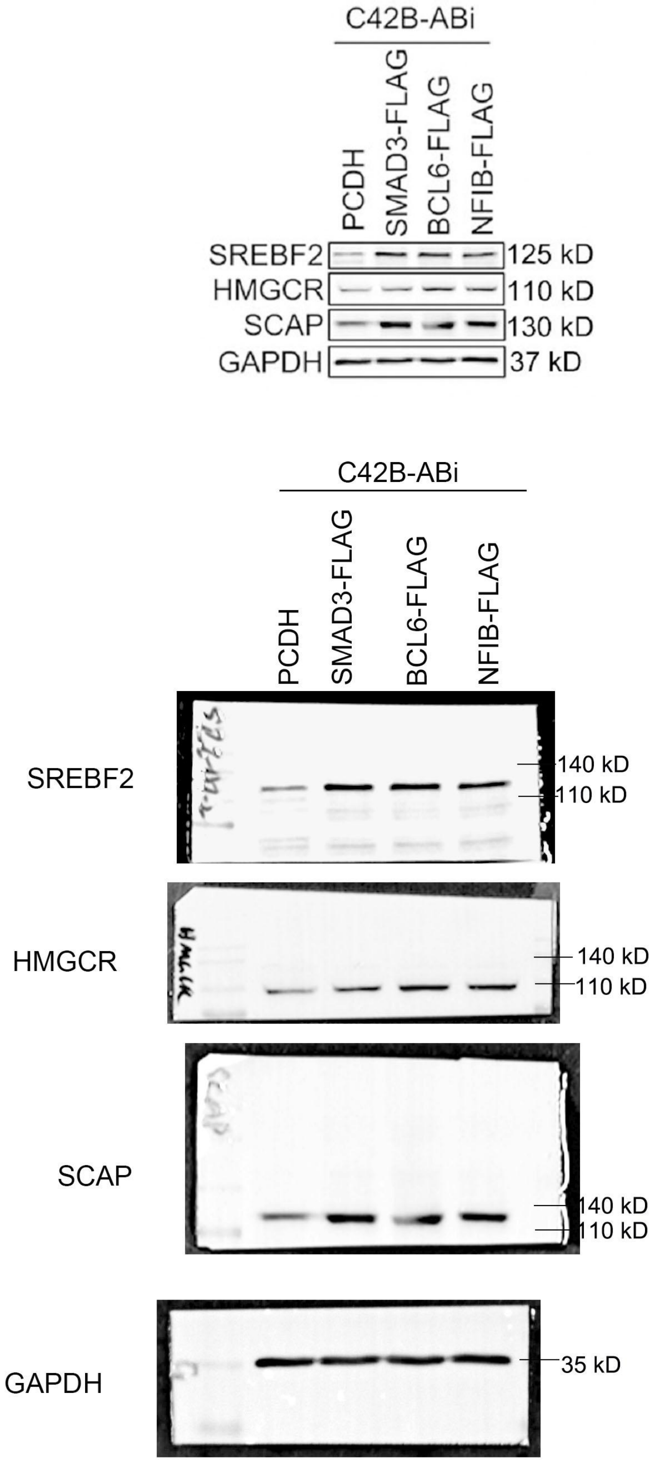

C42B-ABi
PCDH
SMAD3-FLAG
BCL6-FLAG
NFIB-FLAG
SREBF2
125 kD
HMGCR
110 kD
SCAP
130 kD
GAPDH
37 kD
C42B-ABi
PCDH
SMAD3-FLAG
BCL6-FLAG
NFIB-FLAG
SREBF2
140 kD
110 kD
HMGCR
140 kD
110 kD
SCAP
140 kD
110 kD
GAPDH
35 kD

Figure 6F

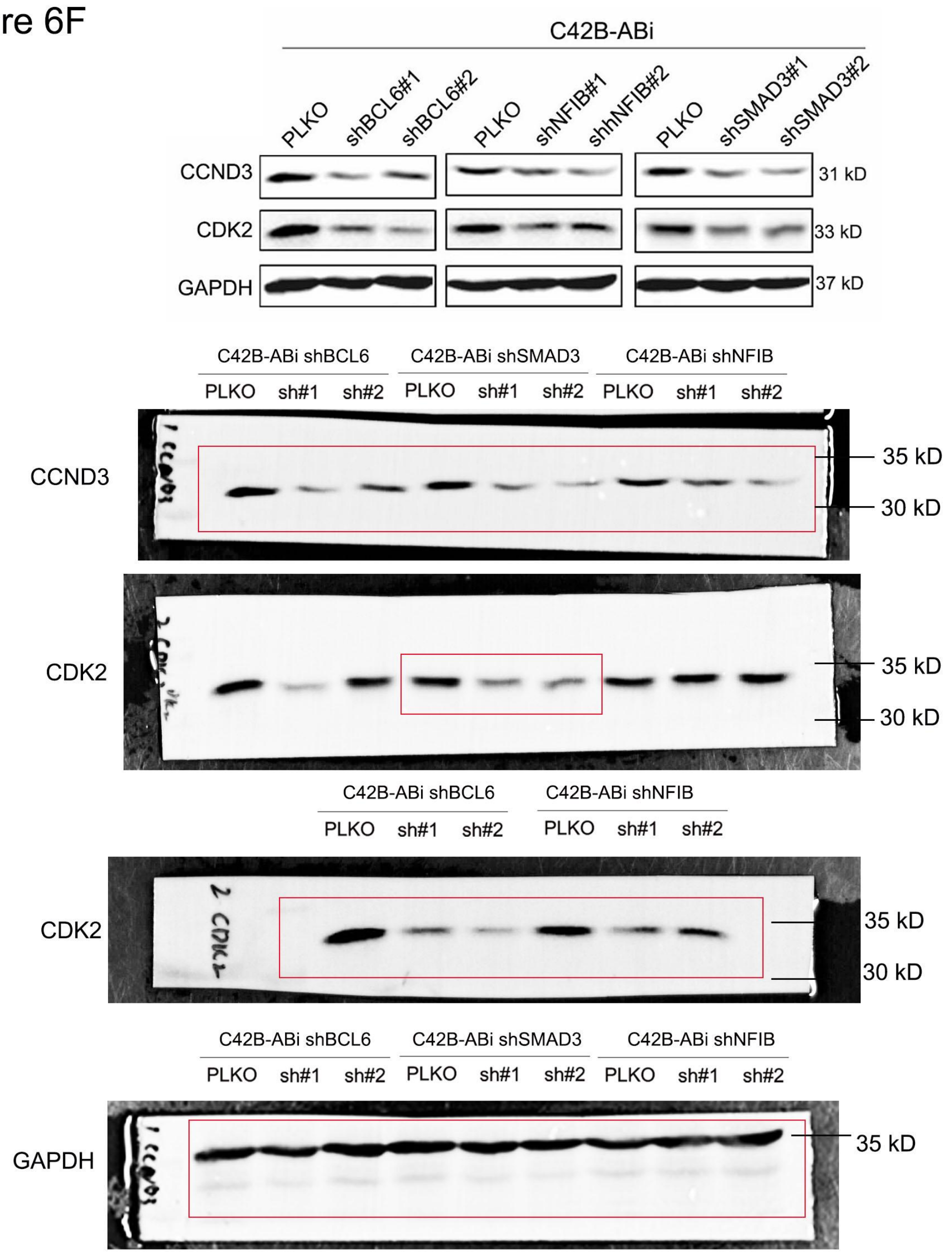

Figure 6G

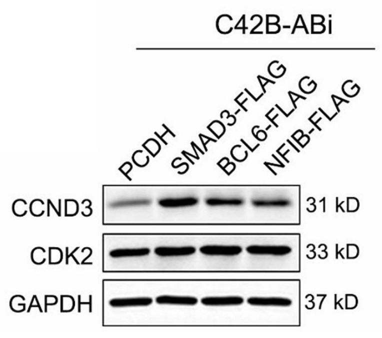

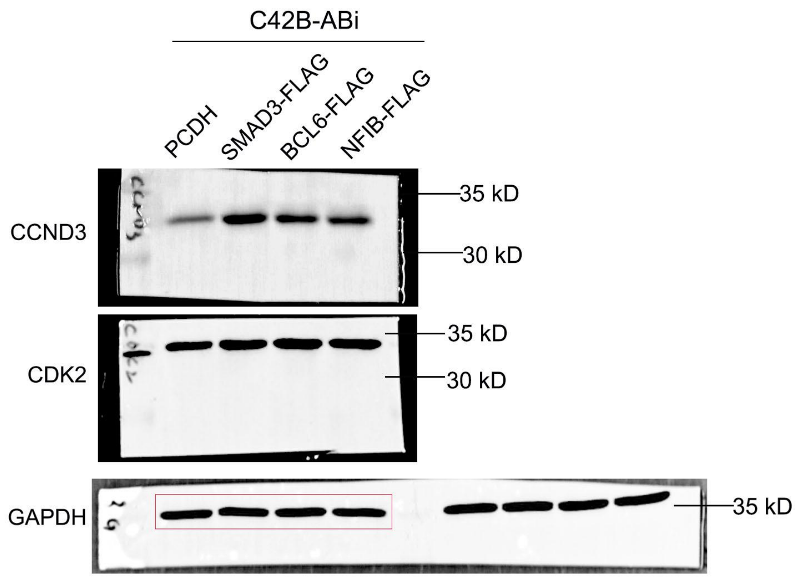

Supplementary Figure 2D

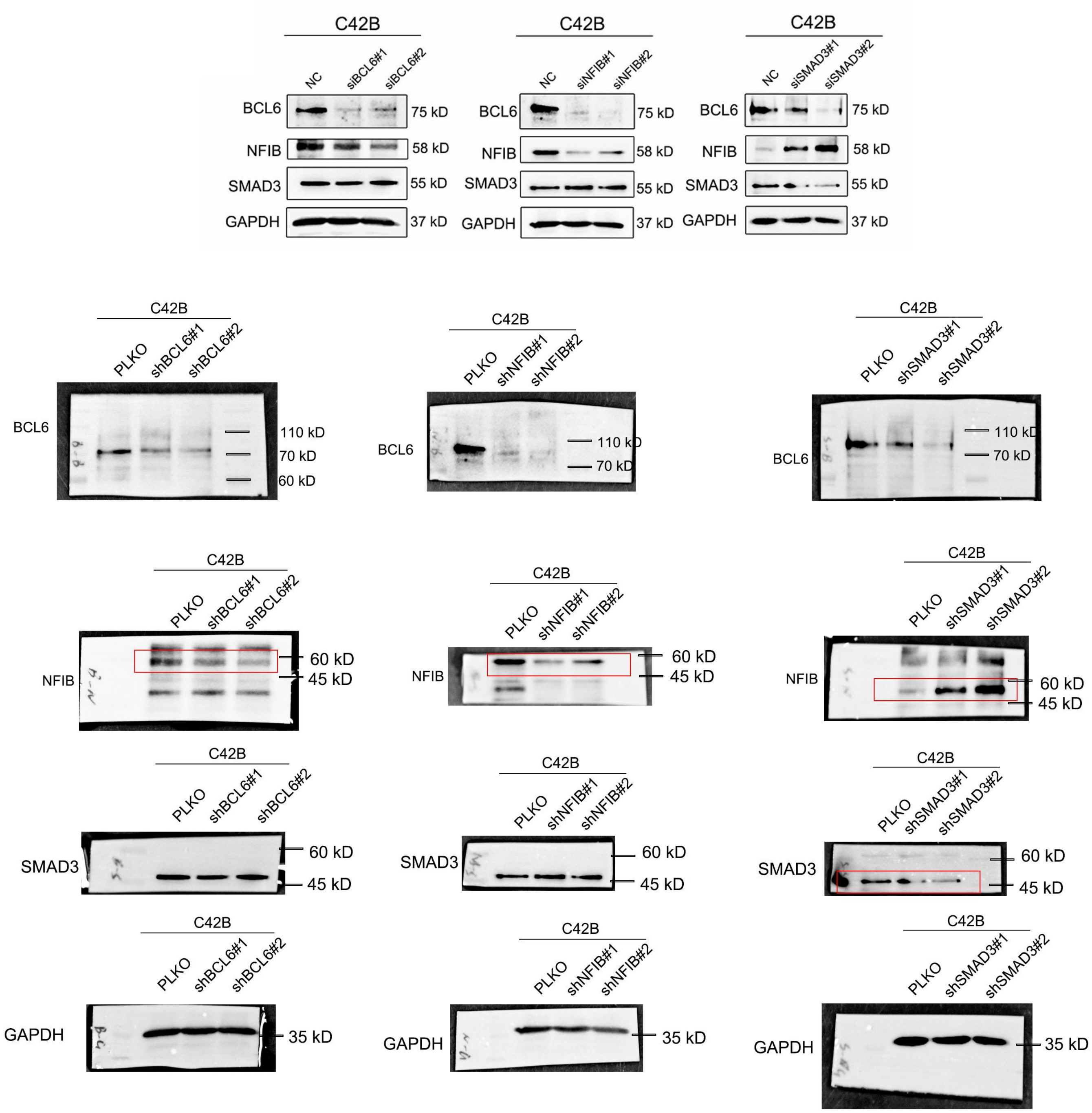

Supplementary Figure 2E

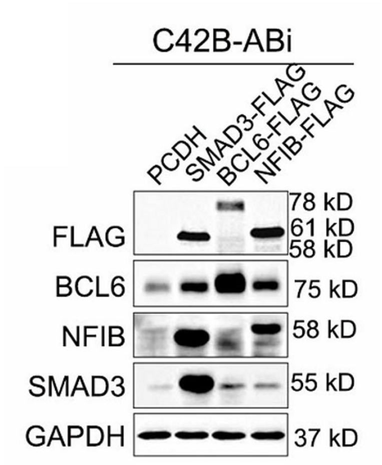

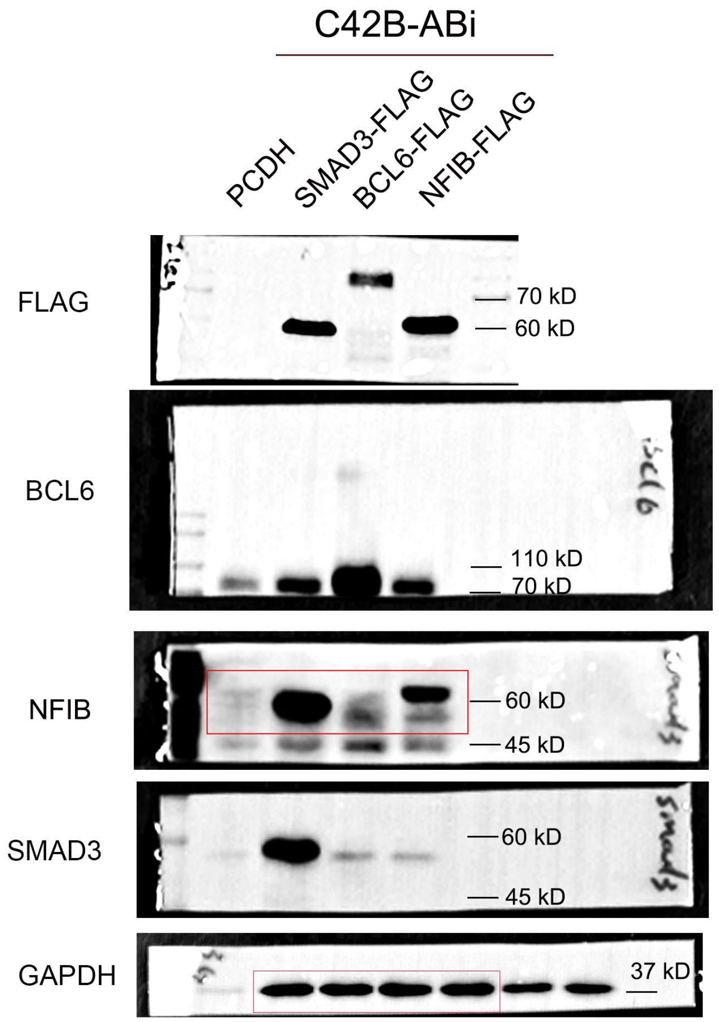

Supplementary Figure 2F

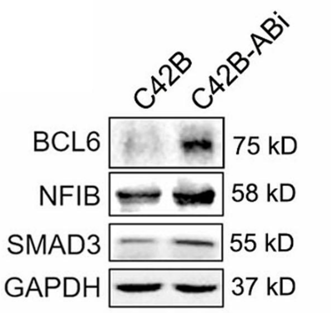

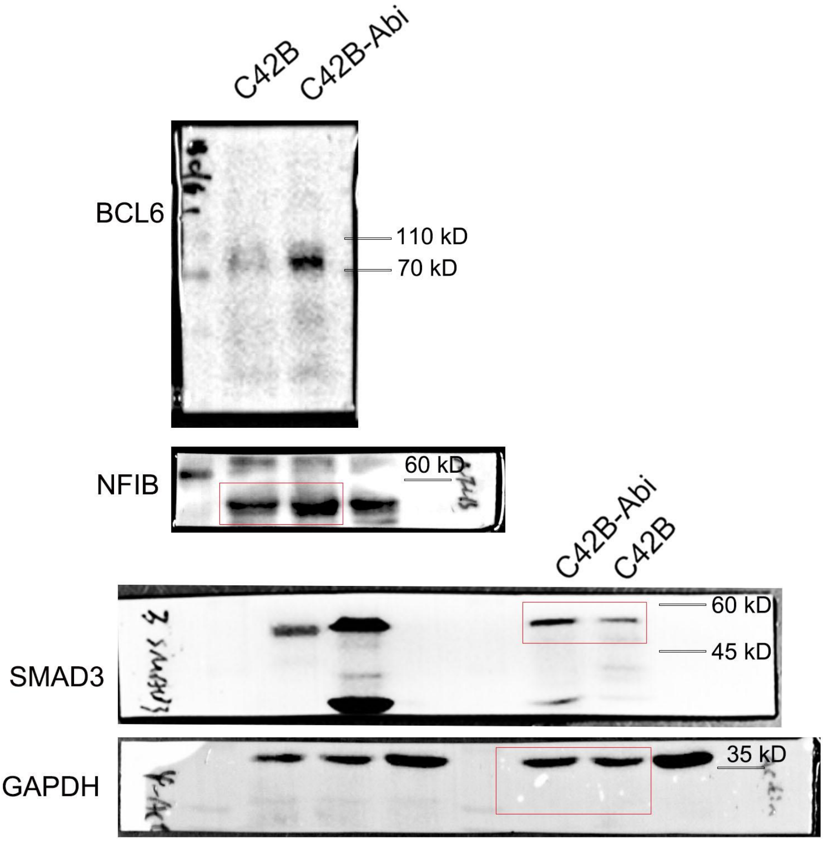

Supplementary Figure 4C

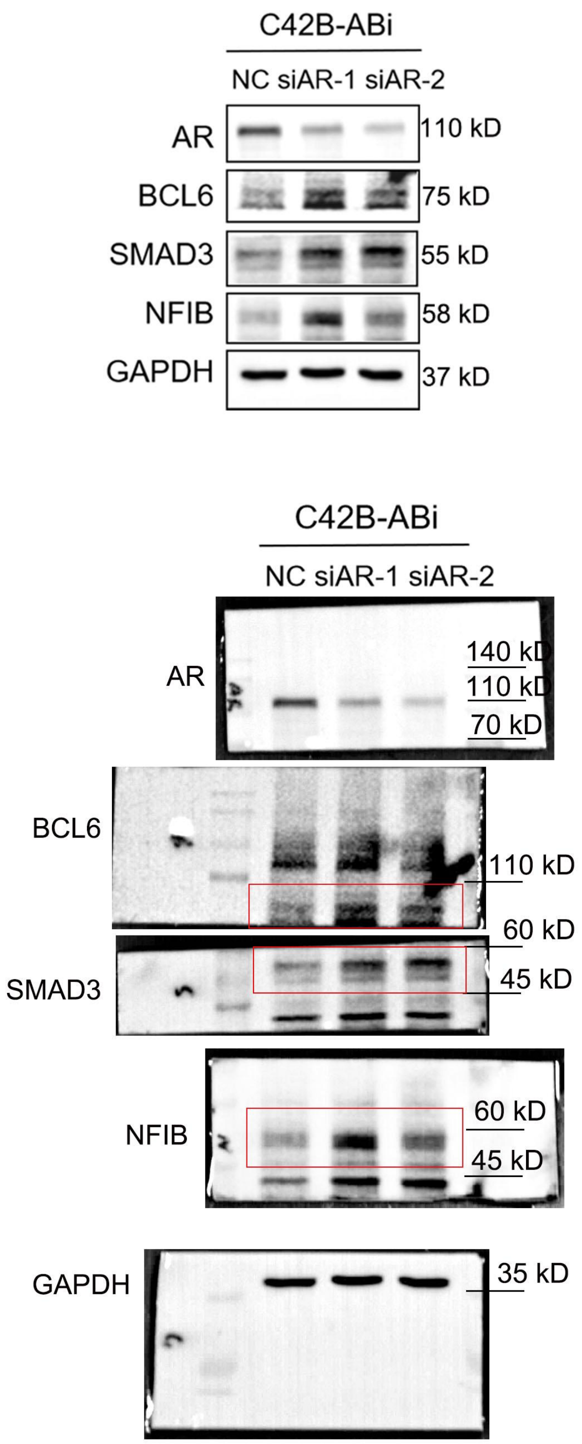

Supplementary Figure 4D

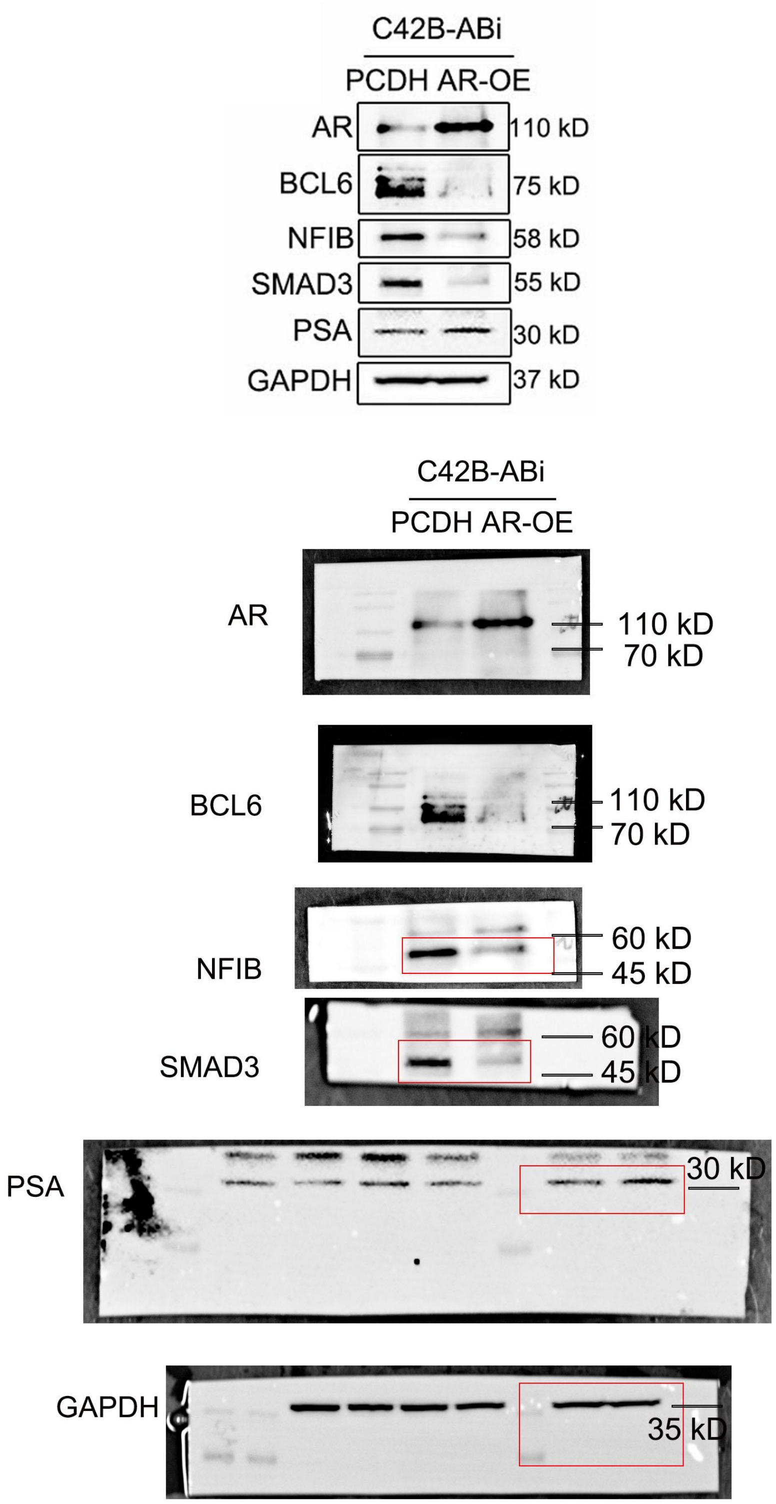

Supplementary Figure 4E

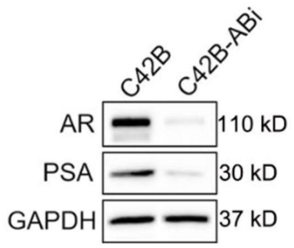

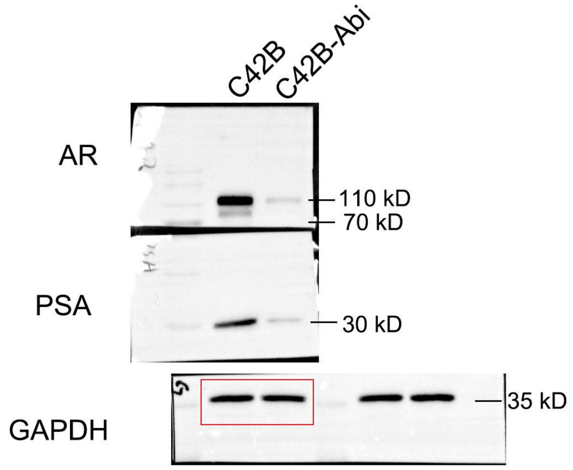

Supplementary Figure 4F

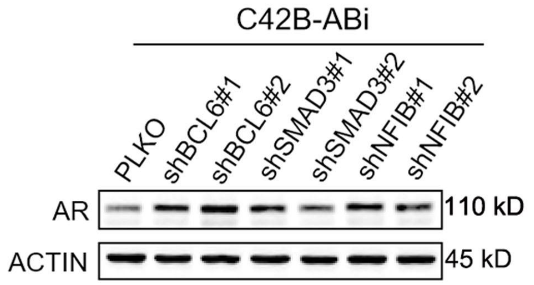

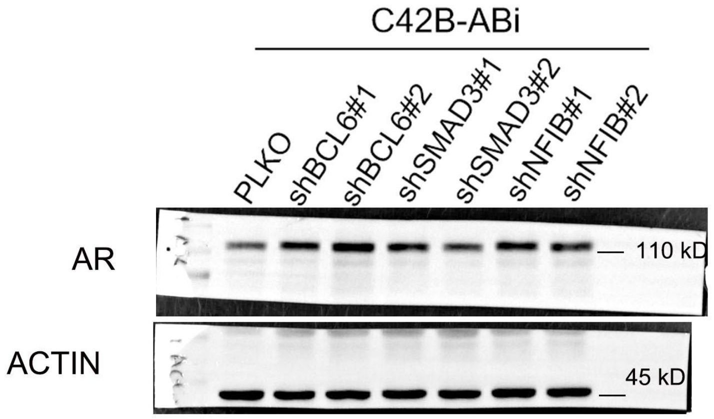

Supplementary Figure 4G

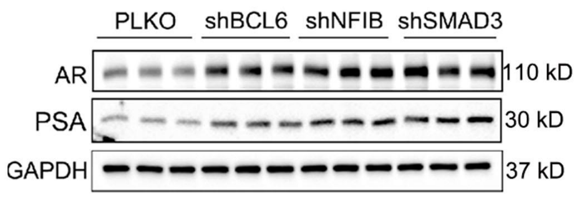

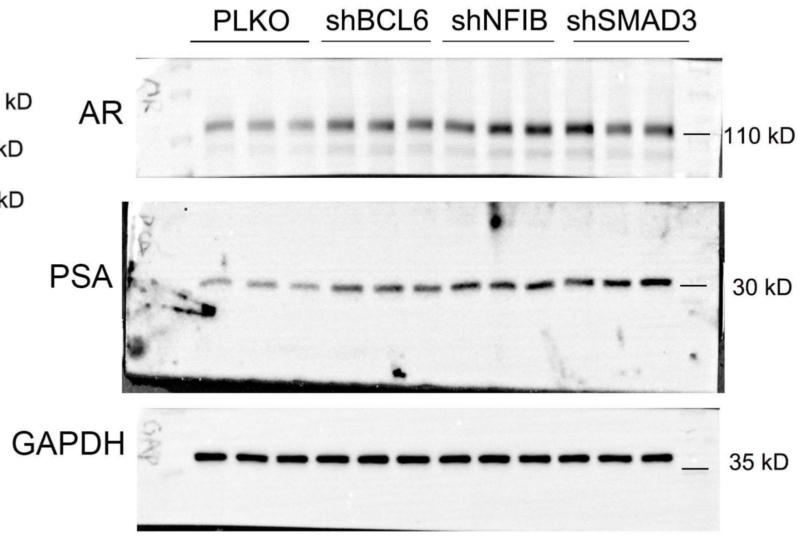

Supplementary Figure 5H

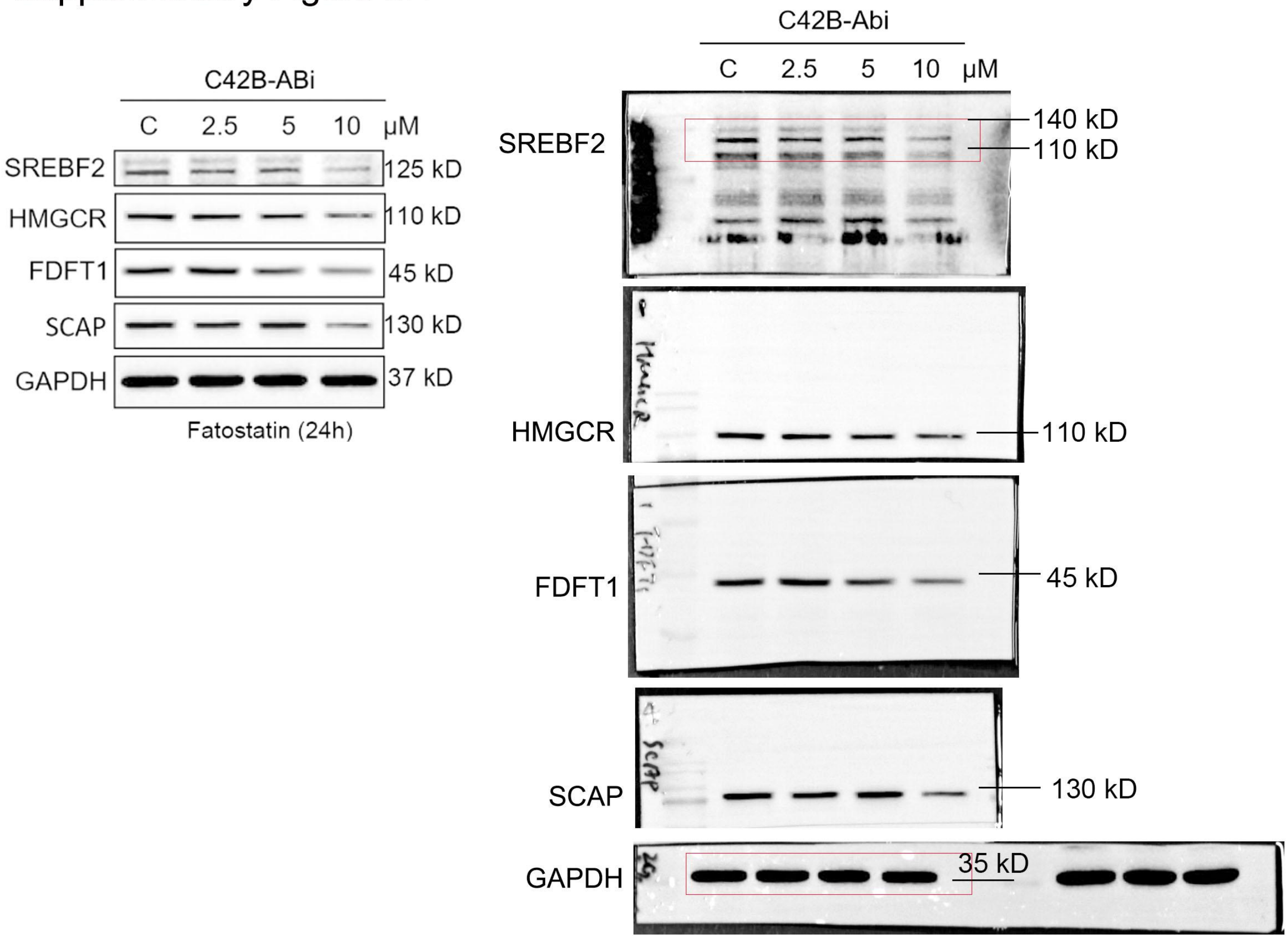

Supplementary Figure 5I

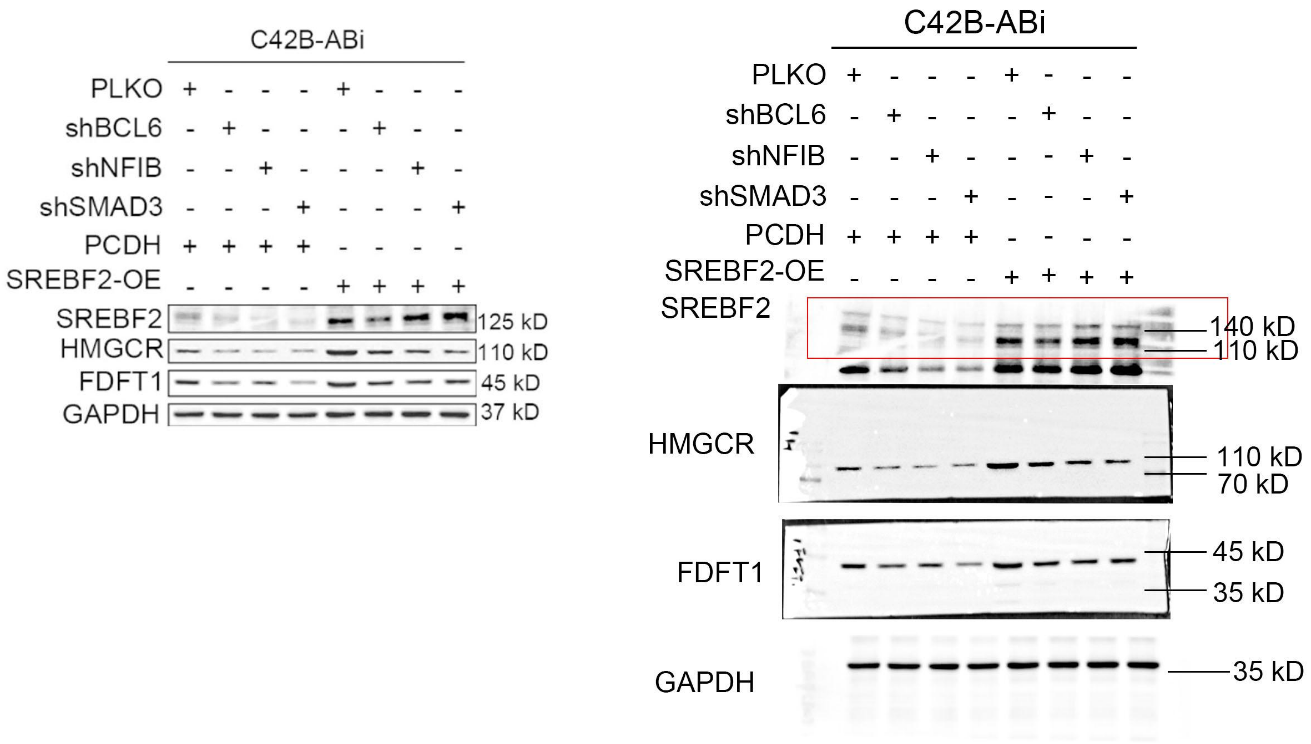

Supplement: Supplementary file 2 — Supporting Information [file ADVS-12-e01284-s003.pdf]
